# The small inhibitor WM-1119 effectively targets KAT6A-rearranged AML, but not KMT2A-rearranged AML, despite shared KAT6 genetic dependency

**DOI:** 10.1186/s13045-024-01610-0

**Published:** 2024-10-08

**Authors:** Mathew Sheridan, Muhammad Ahmad Maqbool, Anne Largeot, Liam Clayfield, Jingru Xu, Natalia Moncaut, Robert Sellers, Jessica Whittle, Jerome Paggetti, Mudassar Iqbal, Romain Aucagne, Laurent Delva, Syed Murtuza Baker, Michael Lie-a-Ling, Valerie Kouskoff, Georges Lacaud

**Affiliations:** 1 Stem Cell Biology Group, Cancer Research UK Manchester Institute, The University of Manchester, Manchester, UK; 2 GSK Medicines Research Centre, Stevenage, UK; 3 Department of Cancer Research, Tumor Stroma Interactions, Luxembourg Institute of Health, Luxembourg, Luxembourg; 4 Genome Editing and Mouse Models, Cancer Research UK Manchester Institute, The University of Manchester, Manchester, UK; 5 Computational Biology Support, Cancer Research UK Manchester Institute, The University of Manchester, Manchester, UK; 6 Division of Informatics, Imaging and Data Sciences, Faculty of Biology, Medicine and Health, The University of Manchester, Manchester, UK; 7 UFR des Sciences de Santé, Inserm U1231, Université de Bourgogne, Team Epi2THM, LipSTIC Labex, Dijon, France; 8 Division of Developmental Biology and Medicine, The University of Manchester, Manchester, UK

## Abstract

**Background:**

The epigenetic factors KAT6A (MOZ/MYST3) and KMT2A (MLL/MLL1) interact in normal hematopoiesis to regulate progenitors’ self-renewal. Both proteins are recurrently translocated in AML, leading to impairment of critical differentiation pathways in these malignant cells. We evaluated the potential of different KAT6A therapeutic targeting strategies to alter the growth of KAT6A and KMT2A rearranged AMLs.

**Methods:**

We investigated the action and potential mechanisms of the first-in-class KAT6A inhibitor, WM-1119 in KAT6A and KMT2A rearranged (KAT6Ar and KMT2Ar) AML using cellular (flow cytometry, colony assays, cell growth) and molecular (shRNA knock-down, CRISPR knock-out, bulk and single-cell RNA-seq, ChIP-seq) assays. We also used two novel genetic murine KAT6A models combined with the most common KMT2Ar AML, KMT2A::MLLT3 AML. In these murine models, the catalytic activity of KAT6A, or the whole protein, can be conditionally abrogated or deleted. These models allowed us to compare the effects of specific KAT6A KAT activity inhibition with the complete deletion of the whole protein. Finally, we also tested these therapeutic approaches on human AML cell lines and primary patient AMLs.

**Results:**

We found that WM-1119 completely abrogated the proliferative and clonogenic potential of KAT6Ar cells *in vitro.* WM-1119 treatment was associated with a dramatic increase in myeloid differentiation program. The treatment also decreased stemness and leukemia pathways at the transcriptome level and led to loss of binding of the fusion protein at critical regulators of these pathways. In contrast, our pharmacologic and genetic results indicate that the catalytic activity of KAT6A plays a more limited role in KMT2Ar leukemogenicity, while targeting the whole KAT6A protein dramatically affects leukemic potential in murine KMT2A::MLLT3 AML.

**Conclusion:**

Our study indicates that inhibiting KAT6A KAT activity holds compelling promise for KAT6Ar AML patients. In contrast, targeted degradation of KAT6A, and not just its catalytic activity, may represent a more appropriate therapeutic approach for KMT2Ar AMLs.

**Supplementary Information:**

The online version contains supplementary material available at 10.1186/s13045-024-01610-0.

## Introduction

Acute myeloid leukemia (AML) occurs when mutations in human hematopoietic stem cells (HSCs) [1, 2] generate leukemic stem cells whose large, immature blast cell progeny cannot differentiate [3], resulting in infiltrative bone marrow failure and death. Overall survival for many patients remains poor [4], necessitating the addition of new treatments to what is a relatively limited therapeutic armamentarium. KAT6A (also called MOZ and MYST3) is an evolutionarily conserved member of the MYST family of lysine acetyltransferases (KATs) [5, 6] that is critical for the development [7] and maintenance of HSCs [8, 9]. The histone/lysine acetyltransferase (HAT/KAT) activity of KAT6A is essential for HSC development [10] and maintenance [11] whilst the co-activation of the hematopoietic specific transcription factors RUNX1 [12] and PU.1 [9] through interaction with KAT6A’s serine/methionine (SM)-rich domain [13] are also likely contributory. KAT6A interacts with the mixed lineage leukemia (MLL) transcription factor/H3K4 methyltransferase (KMT2A) through its catalytic KAT domain to regulate HOXA gene expression in HSCs [14], and is one of the three ‘most significant upstream regulators’ of the HOXA cluster in AML [15].

KAT6A is a genetic vulnerability in KMT2A (MLL) rearranged (KMT2Ar) AML [16–18], a finding corroborated by the DepMap portal [19–21]. Yan et al. [17] showed the importance of acetylation by KAT6A in driving stemness in a human KMT2A::MLLT3 AML cell line. WM-1119, the first-in-class inhibitor of the MYST family acetyltransferases [22], which has entered Phase 1 clinical trials (NCT04606446, trial registered October 28, 2020) in solid-organ cancers, has been proposed as a potential therapy in KMT2A::MLLT3 AML [17].

AMLs in which KAT6A is translocated (KAT6A rearranged, KAT6Ar) have dismal prognoses, with five-year relapse-free survival of 7% extending to only 26% in those allografted in first complete remission and five-year overall survival rates of 11% and only 38%, respectively [23]. Better therapies to prolong survival following relapse are critically needed. Because the KAT domain of KAT6A is retained in the KAT6A-fusion protein created by translocation [5] and the other fusion partners are also often a KAT (CREBBP/CBP/KAT3A [24–28], P300/KAT3B [29, 30], or NCOA2/TIF2/KAT13C [31–33]), such fusions have been postulated to act through a super-KAT activity [34]. Inhibition of KAT activity is, therefore, a logical therapeutic target.

In this work, we investigated the use of WM-1119 in a murine cell line of KAT6Ar AML, hereon referred to as cell line MT2 (*M*OZ::*T*IF*2*/KAT6A::NCOA2, MT2) and KAT6Ar patient samples. We found that WM-1119 completely abrogated the proliferative and clonogenic potential of MT2 cells *in vitro.* WM-1119 treatment was associated with decreased stemness and leukemia pathways at the transcriptome level, related to loss of binding of the KAT6A::NCOA2 fusion protein at critical regulators of these pathways.

To investigate a broader therapeutic role of targeting KAT6A in AML, we employed novel murine KAT6A models combined with the most common KMT2Ar AML, KMT2A::MLLT3 AML [35]. In our KMT2A::MLLT3 AML murine models, the catalytic activity of KAT6A, or the whole protein, can be conditionally abrogated or deleted. We demonstrated with these mouse models and validated in human cell lines that functions of KAT6A beyond its catalytic activity are important for KMT2A::MLLT3 leukemogenesis. Our findings indicate that while inhibiting the KAT activity of KAT6Ar AML patients holds great therapeutic promise, using WM-1119 as a ligand around which to develop targeted degradation may be the most appropriate therapeutic strategy for patients with KMT2Ar AML.

## Materials and methods

### Mice

All procedures involving animals were performed under UK Home Office Project License PP3007645 in accordance with the Animals (Scientific Procedures) Act 1986.

The mouse line carrying a HAT catalytic dead mutant version (Q654E/G657E) of KAT6A (KAT6A^MUT^) was generated by the Genome Editing and Mouse Models Core Facility of the CRUK Manchester Institute by CRISPR-Cas technology. Briefly, a sgRNA (5’- TAGCCCTTACGTTGGTATTGGGG) targeting the region of interest was designed using the Sanger WTSI website http://www.sanger.ac.uk/htgt/wge/ [36]. Complementary oligos carrying the specific 20-nucleotide sequence of the gRNA were annealed and cloned into a vector containing the gRNA backbone and a T7 promoter for in vitro transcription using MEGAshortscript T7 Kit (Life Technologies, AM1354) and purified with MEGAclear Kit (Life Technologies, AM1908). The repair template carrying Q654E and G657E new point mutations was designed as an Ultramer (IDT DNA technologies). To facilitate screening of targeted events by PCR-RFLP, a DdeI restriction silent mutation was inserted in the PAM sequence, to avoid the retargeting by Cas9 endonuclease. Superovulated C57Bl/6OlaHsd 4-weeks old female mice were mated to C57Bl/6OlaHsd stud males, and fertilized embryos were collected from oviducts at 0.5 days post coitum (dpc). Embryos were microinjected in the cytoplasm with mRNA Cas9 (100ng/µl; TriLink, L-T206), in vitro transcribed sgRNA (25ng/µl) and Ultramer ssDNA (100ng/µl) as repair template. Zygotes were cultured overnight, and 2-cell embryos surgically implanted into the oviduct of 0.5dpc Hsd: ICR (CD-1) pseudo pregnant mice. The offspring was assessed for correct targeting using PCR-RFLP primers (Fwd 5’CTTGGTTTTGGTGGCAGGTT and Rev 5’ TGACTCCTCCTGCATGTTGT) flanking the homology arms followed by DdeI restriction digestion (New England Biolabs, R0175S) and subsequent Sanger sequencing.

The conditional *Kat6A* allele with exon 4 flanked by loxP sites (KAT6A^FL^) was produced at the Institute Clinique de la Souris (ICS: Illkirch, France) on commission and design from L Delva’s laboratory. The targeting vector contains a 0.9 kb fragment corresponding to the “floxed” fragment (including exon 4 of Kat6a) together with the 4.3 kb 5’ homologous and the 3’ homologous arms. The linearized construct was electroporated into murine embryonic stem cells with a 129S2/SvPas genetic background. After selection, target clones were identified by PCR using external primers, then confirmed by Southern blots with Neo and external 5’ and 3’ probes (Supplementary Fig S6). A positive murine embryonic stem cell clone was obtained. It was microinjected into blastocysts of C57BL/6J genetic background mice, and chimeric males enabled germline transmission.

Both GEMMS were bred on a C57/Bl6 background and crossed with B6.129-Gt(ROSA)26Sortm1(cre/ERT2)Tyj/J [37], which contains the Cre-ERT2 knocked into the ROSA26 locus, and with mTmG reporter mice [38] generated by injection of ES cells. The CreERT2 fusion protein consists of Cre recombinase fused to a triple mutant form of the human estrogen receptor, which does not bind its natural ligand (17β-estradiol) at physiological concentrations but will bind the synthetic estrogen receptor ligand 4-hydroxytamoxifen.

All mice were housed under specific pathogen-free conditions and in individually ventilated cages maintained at 20-24°C and 40-60% relative humidity. The mice were genotyped by Transnetyx.

### Competitive repopulation

C57Bl/6 CD45.1 Pepc recipient mice were irradiated sub-lethaly (125 Gy) 4 h before cell injection. One million bone marrow cells of the test group (*Kat6a* KO or *Kat6a* WT, C57Bl/6 CD45.2) and of the competitor group (CD45.1.2) were co-injected intravenously. Bone marrow/Peripheral blood reconstitution was evaluated after 15 weeks.

### Generation and culture of murine MT2 (KAT6A::NCOA2) and KMT2A::MLLT3 AML cell lines

C-kit positive cells were sorted from the whole bone marrow of female C57BL/6 or female/male transgenic mice with the desired genotype by magnetic bead selection (Miltenyi Biotec) and then retrovirally transduced with an MSCV-KAT6A::NCOA2 or MSCV-KMT2A::MLLT3 construct. After passage through three to seven rounds of methylcellulose supplemented with 5% foetal calf serum (FCS)(Sigma-Aldrich, F7524), 0.5% L-glutamine (Gibco, 25030081), 0.5% Penicillin/Streptomycin (Gibco, 15140163), 0.5% kit ligand supernatant, 0.5% IL-3 supernatant, 0.5% GM-CSF supernatant, 0.5% thrombopoietin supernatant, 0.3% transferrin supernatant, 0.13% monothiolglycerol (Sigma, M6145), 0.25% ascorbic acid, 0.05% recombinant IL-11 (Pepro Tech, 200-11), 0.1% erythropoietin (Pepro Tech, 100-64), 0.1% IL-6 (Pepro Tech, 216-16) and 0.05% MCSF (Pepro Tech, 315-02), cells were grown under standard conditions (37 °C, 5% CO2) in RPMI-1640 (Fisher Scientific, 21875-091) supplemented with 10% FCS, 1% Kit ligand supernatant, 1% IL-3 supernatant and 10 ng ml^− 1^ recombinant IL-6.

### Mouse models of leukemia

Female wild-type C57BL/6 mice aged 8–12 weeks (Envigo) were given acidified water for 1 week prior and 2 weeks following irradiation with 600 cGy in two divided doses 3 h apart. Following irradiation that same day, cKit positive murine bone marrow retrovirally transfected with an MSCV-KAT6A::TIF2/NCOA2 or MSCV-KMT2A::MLLT3 plasmid were iv injected. Alternatively, splenic cells harvested from primary AML, treated with 4-hydroxytamoxifen (Sigma, T176) and FACS sorted were injected intravenously.

### Human cell lines and cell culture

Human AML cell lines, confirmed to be mycoplasma free (Venor GeM gEP Mycoplasma Detection Kit, Minerva Biolabs), were authenticated by STR analysis, and grown under standard conditions in RPMI-1640 supplemented with 10% FCS and 1% Penicillin/Streptomycin.

### Primary AML cell culture

Primary AML cells were cultured with MS5 feeder cells in Alpha-MEM supplemented with 25% heat inactivated foetal bovine/calf serum, 25% inactivated horse serum, 50µM β-mercaptoethanol, 1µM hydrocortisone, 1% Penicillin/Streptomycin, 1% L-Glutamine and 20ng/mL each of recombinant human hSCF, hG-CSF, hIL-3, hTPO (all Pepro Tech). 3-5 × 10^5 MS5 feeder cells were cultured the day before thawing of primary cells in 2mL co-culture medium per well of a 6 well plate to which 2mL sterile gelatine had been added for up to 30 min prior to aspiration. The next day, thawed primary cells were carefully added at 1 × 10^6/mL in co-culture and monitored daily to maintain high density. Feeder cells were changed at least twice a week and when primary cells were passaged, 20% conditioned media was maintained. For drug assays, primary cells were initially seeded at 1 × 10^5/mL in a 24 well plate (1mL/well) in 20% conditioned co-culture media from MS5 cells.

### Retrovirus production and transduction

4.5 million platinum E cells were seeded in 10mLs of RPMI media in 10 cm dishes. Approximately 16 h later, 7 µg of the retroviral vector of interest was mixed with 42 µg polyethyleneimine in 1mL serum-free RPMI and added to the cells. Media was collected after 48 h, filtered and added at 500µL per 25,000 cKit positive cells with 8 µg/ml Hexadimethrine bromide (Sigma, 107689) and spun for 45 min at 480 g at 32 °C.

### Lentivirus production and transduction

HEK 293T cells were seeded into 10 cm diameter dishes at between 3 and 5 million cells per dish. The following day, cells were transfected with 4 µg of the lentiviral vector of interest (shRNA or guide) and 2nd generation lentiviral packaging vectors using 42 µg polyethyleneimine in 1 ml serum-free DMEM. The next day the media was changed, and the viral supernatant was harvested on the subsequent day. For transductions, cells were resuspended in viral supernatant supplemented with 8 µg/ml Hexadimethrine bromide (Sigma, Cat. No. 107689) and transferred to multi-well plates. Cells were centrifuged at 480 *g* for 30 min at 32 °C and incubated in viral supernatant at 37 °C for 24 h.

### Cell proliferation assays

2 × 10^5^ MT2 cells per ml were treated with doses of WM-1119 (Biotecne, Kyiv) as detailed or DMSO (volume equivalent to that within highest WM-1119 dose). Cells were split 1:3 every 48 h and drug/vehicle was replenished. Cell aliquots were counted using a haemocytometer or evaluated through Cell Titer Glo^®^ (Promega) analysis, performed as per manufacturer’s instructions.

### Clonogenic assays in methylcellulose

Murine AML cells were plated in duplicate or triplicate at a cell dose of 5 × 10^2^ per ml in cytokine supplemented methylcellulose (see above) in the presence of vehicle (0.1% DMSO) or 4-hydroxytamoxifen **(**25nM for MT2 cells and 50nM for KMT2A::MLLT3 cells in round 1 and 10nM from round 2 onwards for both cell lines). Cells were incubated at 37 °C and 5% CO_2_ for 7–10 days, and colonies and total cell numbers were counted.

### shRNA

shRNA’s were designed using the sequential learning algorithm SplashRNA [39] available at http://splashrna.mskcc.org/ and were cloned into LT3GEPIR (Addgene plasmid #111177, Johannes Zuber). ShRNA constructs were lentivirally transfected into human AML cell lines and underwent puromycin selection. ShRNA (and GFP) expression induction was activated by the addition of 1 µg doxycycline to 1mL media.

### CRISPR cell line generation

THP-1 cells expressing Cas9 were a gift from Ricky Johnstone (Peter MacCallum Cancer Center, Australia). NOMO-1 Cas9 cells were generated by lentiviral transduction with virus generated with the plasmid LentiV_Cas9_puro (Addgene plasmid #108100 from Christopher Vakoc) and subsequently selected with puromycin. Cas9 efficiency was confirmed by CRISPR mediated cellular competition assays using guides against essential human genes (*CDK9*,* CDK1*,* PCNA*,* RPA3*) and a guide against a murine control gene (*Rosa*).

### Proliferation and differentiation of CRISPR KO of KAT6A

Single guides were cloned into a derivative of pCRISPRia-V2 (Addgene plasmid #84832, Jonathan Weissman) expressing BFP. Cells were split every 2–3 days and the percentage of transduced BFP-positive (KO) cells was monitored by flow cytometry. Values were normalised to the BFP-positive percentage on day 4. For differentiation analyses, cells were harvested from the well of a 6-well plate, and 1mL of pre-warmed detachment buffer (1xPBS, 0.144 g glucose, 4.8mL EDTA (0.5 M) to an 800mL final volume) was added and incubated for two minutes before addition to the harvested cells. All cells were spun, washed in PBS and blocked with CD16/CD32 monoclonal antibody (eBioscience) for 10 min prior to staining with CD11b monoclonal antibody (ICRF44) APC and CD86 monoclonal antibody (IT2.2) PE-Cy7 (both eBioscience) both at 1:200 dilution. The cells were analysed for BFP, CD11b and CD86 on a BD Fortessa X20 (BD Biosciences).

### Cytospin

Cells were cytocentrifuged onto slides using a ThermoFisher Shandon 3 cytospin centrifuge at 600 rpm, medium acceleration for 5 min and were then fixed in May Grunwald for 5 min and stained in Giemsa for 20 min.

### Flow cytometric analyses

Flow cytometry analyses were conducted with a BD Fortessa X20 (BD Biosciences). Fluorescence-activated cell sorting (FACS) was performed on BD Aria III (BD Biosciences). Data were analyzed using FlowJo v10 software.

### Analysis of cell cycle

The cell cycle was analyzed by flow cytometry using propidium iodide to measure DNA content. Briefly, cells were pelleted and washed once with cold PBS. Cell pellet was resuspended in 70% ethanol and incubated at 4 C for 30 min to fix and permeabilise the cells. Fixed cells were then washed twice with cold PBS and resuspended in PBS containing 5 µg/mL RNAse for 30 min. Finally, propidium iodide was added to a final concentration of 10 µg/mL. Stained cells were then analysed by flow cytometry.

### qPCR

RNA extracted using RNeasy Plus Mini kit (Qiagen), as per manufacturer’s instructions. cDNA was generated using High-capacity cDNA kit. Gene expression was quantified by qPCR using FastStart Universal Sybr Green Master (Rox) (Roche, Cat. # 04913850001) on a Quantstudio 5 Real-Time PCR system (Applied Biosytems, Cat. # A28140). The standard Comparative Ct with melt program was used. Knockdown was determined using the delta delta Ct method, using GAPDH and U6 as normalizing genes.

### RNA-Seq

RNA was extracted using RNeasy Plus Mini kit (Qiagen), as per manufacturer’s instructions and paired-end Sureselect (Agilent) libraries prepared for sequencing. Libraries were sequenced using the Novaseq (Illumina) platform. Basecalls were converted to FASTQ files using bcl2fastq (Illumina). Fastq files were trimmed with automatic adapter detection using trim galore (version 0.6.10) and aligned to GRCm38 (Ensembl 75) using Star aligner (version 2.5.1b) [40]. BAM alignments were quantified in R (version 4.2.0) using featureCounts from the Rsubread library (version 2.8.2) [41]. DE analysis was performed using DESeq2 (version 1.38.3) [42].

Single cell RNA-seq was performed using Chromium NextGEM Single Cell 3’ v2 reagent kit as per manufacturer instructions (10X Genomics). CellRanger v3.0.2 (10X Genomics) was used to convert raw basecalls to FASTQ, to map reads to GRCm38, to assign reads to cells, and to count reads aligned to each feature. Count data were further analysed in R using Seurat package [43].

### ChIP-Seq

Cells were crosslinked with formaldehyde for 10 min then quenched with glycine. The cells were then lysed, the nuclei pelleted down by centrifugation and sonicated using a Bioruptor Pico (Diagenode). An aliquot was used for fragmentation analyses, and the rest was frozen until use in ChIP assays. Protein-G coated Dynabeads were incubated at 4 °C with Ty1 antibody (MAb-054-050, Diagenode) to prepare beads pre-coated with Ty1 antibody. Sonicated chromatin was added to Ty1 pre-coated beads and the mix was incubated overnight at 4 °C and then washed. Immunoprecipitated chromatin was eluted by two sequential incubations and then reverse-crosslinked for 12 h, followed by treatment with RNase A and Proteinase K and purification of DNA. Purified DNA was quantified with Qubit DS DNA HS Assay (ThermoFisher Scientific, USA).

At least 1ng of ChIP DNA was used to prepare sequencing library with NEBNext Ultra II DNA Library Prep Kit for Illumina (NEB, USA). Indexed libraries were prepared using the MicroPlex Library Prep kit v2 (Diagenode) or the NEBNext Ultra II DNA Library Prep Kit for Illumina (New England BioLabs). Library quality was checked using the Agilent Bioanalyzer. Libraries were quantified by qPCR using the KAPA Library Quantification Kit for Illumina (Kapa Biosystems Inc.). Paired-end 75 bp sequencing was carried out by clustering 1.5pM of the pooled libraries on a NextSeq 500 sequencer with High Output v2 chemistry (Illumina inc.) for MicroPlexed samples. Paired-end 40 bp sequencing was carried out by clustering 1.8pM of the pooled libraries for NEBNext Ultra on a NextSeq 500 sequencer with High Output v2.5 chemistry (Illumina inc). Basecalls were converted to fastq files using bcl2fastq (Illumina). Fastq files were trimmed with automatic adapter detection using trim galore (version 0.6.10) and aligned to GRCm38 (Ensembl 93) using bowtie2 (version 2.5.1) [44]. BAM files were subset to uniquely mapping reads and deduplicated using picard tools (version 1.9.6) [45]. Alignments to ENCODE blacklist regions [46] were filtered from the BAM files and converted to bigwig format. QC was performed on the final alignments using ngsplot and deeptools (versions 2.6.3 & 3.5.2). ChIP peaks were called using MACS2 (version 2.2.8) [47]. Comparisons between peaksets were performed in R (version 4.2.3) using functions from the GenomicRanges package [48]. Grouped differential enrichment tests within peaks were performed using DiffBind [49]. Comparison of “peak-ranges” between groups was performed by combining peak widths within 2000 bp of each other and merging between samples; the length of peak-ranges overlapping either over(up)/under(dn)-enriched genes, assessed by DiffBind, were compared.

### Data sharing

Raw sequencing files and processed data are available in GEO (PRJNA1100482).

### Quantification and statistical analyses

Pre-ranked gene set enrichment analysis was performed with GSEA v 4.3.2 software [50]. Genes were ranked by log2 fold change in expression.

Statistical analyses were performed using GraphPad Prism version 10.

## Results

### Inhibiting the catalytic activity of KAT6A impairs the growth of KAT6A translocated AML

**Fig. 1:**
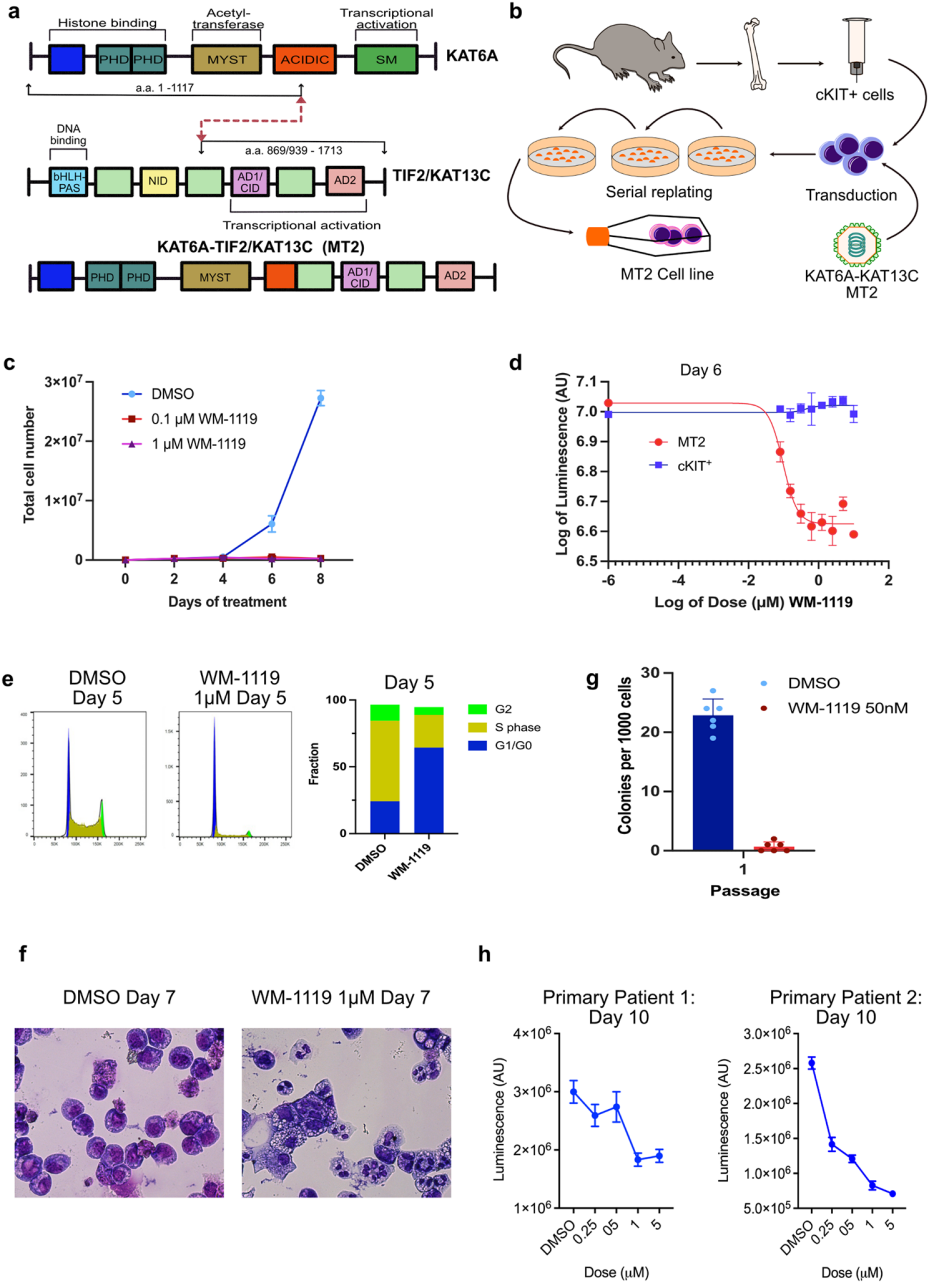
Inhibiting the catalytic activity of KAT6A impairs the growth of KAT6A translocated AML. **a**, Schematic outline of KAT6A and NCOA2 proteins showing known functional domains and the KAT6A::NCOA2 fusion protein. PHD, plant homeodomain. MYST, ‘MOZ, YBF2, SAS2, TIP’ domain. SM, serine-methionine rich domain. bHLH-PAS, basic helix–loop–helix/Per-ARNT-SIM domain. NIP, Nuclear Receptor Interaction Domain. CID, CREB-binding protein Interaction Domain. **b**, Schematic of the generation of the murine KAT6A::NCOA2 AML cell line (MT2). **c**, Cell counts for MT2 cells treated with DMSO and 0.1µM and 1µM WM-1119 every 2 days (*n* = 2, error bars represent mean with standard deviation). **d**, Proliferation (directly proportional to luminescence of Cell-Titer Glo^®^) of MT2 and cKit positive murine cells after treatment with different concentrations of WM-1119 (*n* = 3, error bars represent mean with standard deviation). **e**, Flow cytometric cell cycle analysis of MT2 cells after treatment with DMSO or 1µM WM-1119 for 5 days. **f**, Cytospin images of MT2 cells following DMSO or 1µM WM-1119 treatment. **g**, Colony forming assay for MT2 cells treated with DMSO or 50nM WM-1119 (*n* = 6, error bars represent mean with standard deviation). **h**, Proliferation (directly proportional to luminescence of added Cell-Titer Glo^®^ reagent) of two KAT6A::CREBBP translocated patient samples, AML-24758B and AML-27,420 A treated with DMSO or different doses of WM-1119 for 10 days

Chromosomal recombinations resulting in KAT6A rearranged (KAT6Ar) AMLs are recognised events in AML. Typically, KAT6A is fused with CREBBP (KAT3A/CBP) [23] but cases in which the fusion is between KAT6A and NCOA2 (TIF2/KAT13C), which can recruit CREBBP via its CBP-binding protein Interaction Domain (CID) (Fig. 1a), have been reported [31–33]. These fusions retain and combine the KAT activities of both proteins and have been postulated to deregulate myeloid differentiation through the creation of a super-KAT activity [34]. Inhibition of KAT6A KAT activity could, therefore, be a logical therapeutic option. To evaluate the relevance of targeting KAT6A KAT activity, we first generated a murine KAT6Ar AML cell line by transducing c-Kit^+^ hematopoietic stem and progenitor cells (HSPCs) with a retrovirus encoding KAT6A::NCOA2 and serially replating the transduced cells to generate a KAT6A::NCOA2 (MOZ::TIF2) cell line hereon referred to as MT2 cells (Fig. 1b). We then evaluated the relevance of the KAT6A KAT activity with the KAT6A KAT inhibitor WM-1119. Treatments of the MT2 cells with low doses of WM-1119 lead to complete abrogation of their proliferation in culture (Fig. 1c). In contrast, there was no WM-1119 effect on the proliferation of untransformed c-Kit^+^ cells at these doses, suggesting a therapeutic window (Fig. 1d). The WM-1119-induced block in MT2 proliferation was accompanied by a marked reduction of cells in S and G2 phases (Fig. 1e) and increased morphological and FACS evidence of differentiation, indicating a release of the myeloid differentiation block pathognomonic of AML (Fig. 1f, Supplementary Fig S1). WM-1119 also rapidly arrested the clonogenic replating capacity of MT2 cells (Fig. 1g). Primary human patient samples harbouring the KAT6A::CREBBP (MOZ::CBP) rearrangement were sensitive to WM-1119, with a substantial reduction in proliferation measured by Cell-Titer Glo after ten days of treatment with low doses of WM-1119 (Fig. 1h). These findings demonstrate significant impacts of WM-1119 on the growth and myeloid differentiation of KAT6Ar AMLs.

### WM-1119 abrogates KAT6A::NCOA2 fusion protein binding at genes implicated in leukemogenesis and stemness

**Fig. 2:**
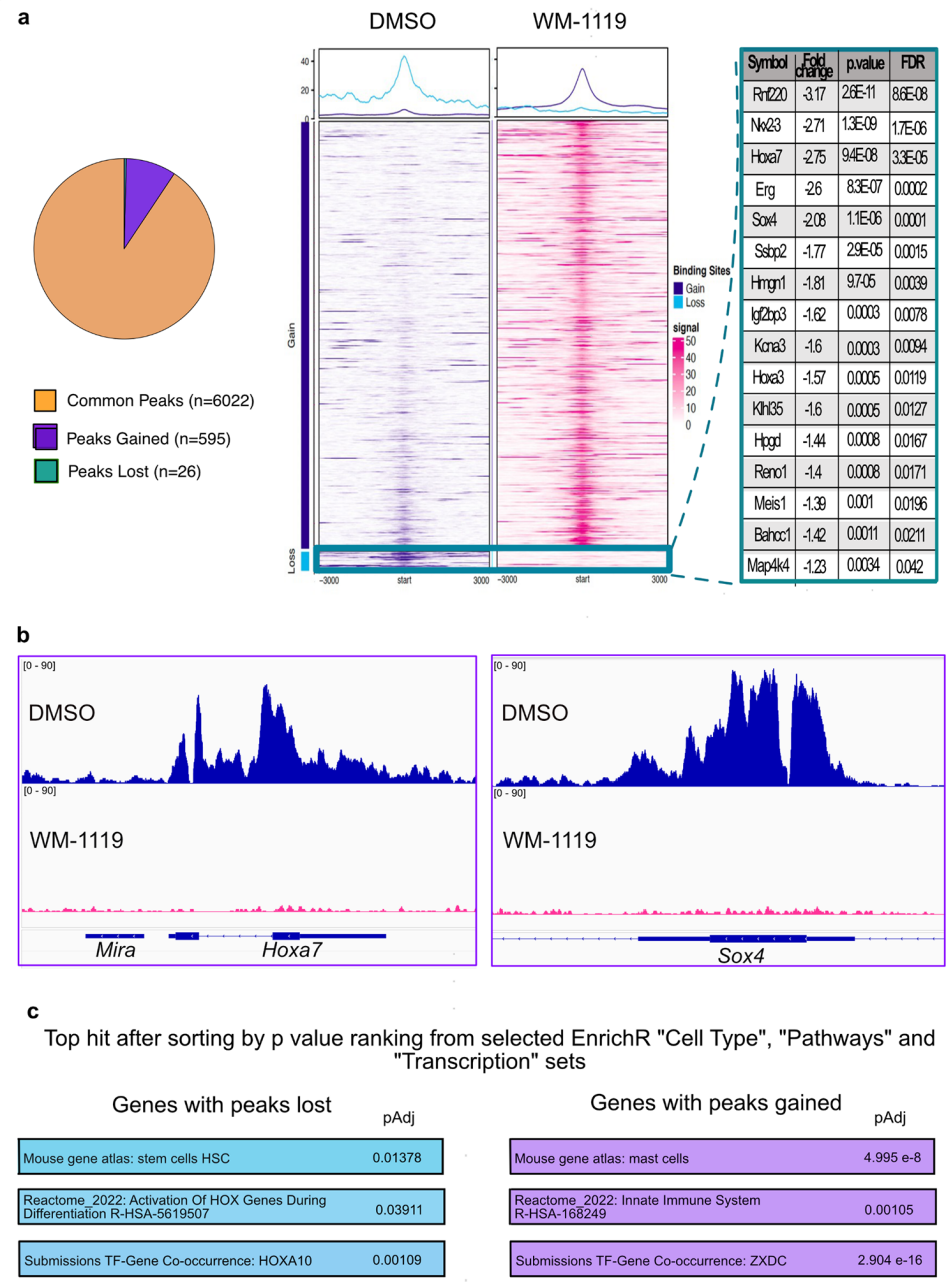
WM-1119 abrogates KAT6A::NCOA2 fusion protein binding at genes implicated in leukemogenesis and stemness. **a**, Pie chart and heat maps showing changes in binding of KAT6A::NCOA2 after treatment with 2 µM WM-1119 compared to DMSO for 72 h. 16 genes, shown in table, show a loss of KAT6A::NCOA2 binding after treatment with WM-1119. **b**, Representative ChIP-Seq histogram tracks showing binding of KAT6A::NCOA2 across the gene bodies of Hoxa7 and Sox4 after 72 h of DMSO treatment and loss of this binding after treatment with 2µM WM-1119 for 72 h. **c**, Top hits by adjusted p value for selected “Cell Type”, “Pathways” and “Transcription” gene sets from EnrichR for genes with loss or gain of KAT6A::NCOA2 binding

We next performed ChIP-Seq using the Ty1 tag present on the KAT6A::NCOA2 construct to investigate whether the phenotypic changes observed upon treatment with KAT6A inhibitor are associated with changes in binding of the KAT6A::NCOA2 fusion protein. KAT6A::NCOA2 binding in MT2 cells was mainly observed at promoters (Supplementary Fig S2a-c). We found that 3 days of treatment with WM-1119 resulted in loss of binding at only 26 sites, whereas 595 peaks were gained and more than 6,000 were maintained (Fig. 2a and Supplementary Fig S2d). Motif analysis using HOMER on all, or just the gained, peaks revealed a bias for CpG (Supplementary Fig S2e), consistent with KAT6A binding to unmethylated CpG islands through its N-terminal winged helix (WH) domain [51, 52]. This N-terminal WH domain is conserved in the protein produced by the translocated KAT6A::NCOA2 cDNA. We also found an interesting, but non-statistically significant, motif enrichment for Myb in the few lost peaks (Supplementary Fig S2e). We concluded from these data that KAT6A::NCOA2, like KAT6A, binds to unmethylated CpG islands of active promoters. The lost peaks were associated with 16 genes, including *Bahcc1*, *Meis1*, *Hoxa3*, *Hoxa7*, *Erg* and *Sox4* (Fig. 2a and b and Supplementary Fig S3). All these genes have been strongly associated with leukemia and stemness. *BAHCC1* is highly expressed in human acute leukemia and represses genes involved in tumor suppression and cell differentiation [53]. The *HoxA* cluster and *Meis1* encode self-renewal hematopoietic transcription factors whose dysregulation has long been recognised in AML [54]. *Erg*, an ETS transcription factor associated with maintaining hematopoietic stem cells (HSCs) in normal hematopoiesis, is highly expressed in most KMT2A::MLLT3 AML [55]. *Sox4*, another potential regulator of HSC activity, is highly expressed in many cancers [56]. Accordingly, EnrichR [57–59] analyses indicated that the genes associated with peaks lost are related to HSCs whereas the genes associated with peaks gained are related to myeloid cells, consistent with differentiation (Fig. 2c, Supplementary Fig S4 and Supplementary Tables 1 and 1). Altogether, these data indicate that treatment of MT2 cells with WM-1119 results in loss of KAT6A::NCOA2 binding at genes associated with leukemia and stemness.

### WM-1119 abolishes the expression of genes implicated in leukemogenesis and stemness

**Fig. 3:**
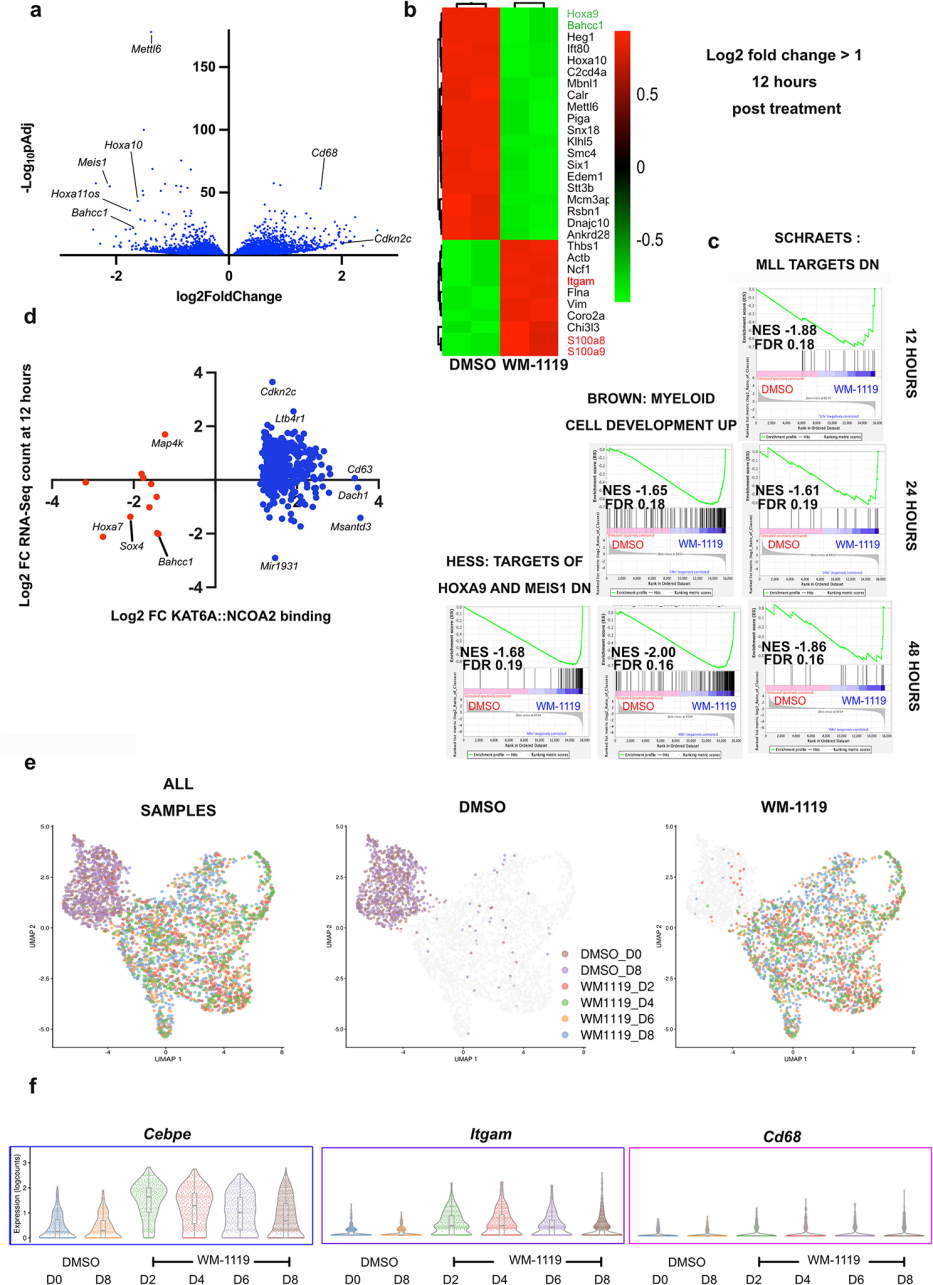
WM-1119 abrogates the expression of genes implicated in leukemogenesis and stemness. **a**, Volcano plot showing differential gene expression in MT2 cells treated with 1µM WM-1119 compared to cells treated with DMSO at 48 h. **b**, Heatmap of genes with Log2 fold change ≥1 at 12 h after treatment. **c**, Leading edge plots for selected gene sets with FDR < 0.25 enriched in bulk MT2 cells 12, 24 and 48 h after treatment with 1µM WM-1119. **d**, Comparison of differential gene expression in cells treated with 1µM WM-1119 compared to cells treated with DMSO at 12 h for genes with loss or gain of KAT6A::NCOA2 binding at 72 h following treatment with 2µM WM-1119 compared to DMSO. **e**, UMAP clustering and **f**, violin plots for selected markers of myeloid differentiation of single MT2 cells at days 0 and 8 after treatment with control (DMSO) and at days 2, 4, 6 and 8 after treatment with 1µM WM-1119

To define further the consequences of WM-119 treatment, we performed a two-day treatment time course (12, 24 and 48 h) on MT2 cells. Significant alterations in gene expression were observed within 48 h (Fig. 3a). Specifically, the leukemia/stemness-related genes *Hoxa9* and *Bahcc1* exhibited downregulation as early as 12 h in WM-1119-treated cells compared to those treated with DMSO. Conversely, *Itgam*, which encodes the myeloid differentiation marker CD11b and *S100a9* and *S100a8*, encoding two calcium-binding proteins of the neutrophil-rich calprotectin complex, were upregulated (Fig. 3b). Analysis of bulk transcriptomic data at different time points with gene set enrichment analysis (GSEA) revealed an early increase in the expression of genes typically downregulated by the key leukemia/stemness driver KMT2A (SCHRAETS: MLL_Targets_DN), followed by the expression of myeloid cell development genes (BROWN: Myeloid_Cell_Development_UP), and of genes downregulated by HOXA9 and MEIS1 (HESS: Targets_Of_HOXA9_and_MEIS1_DN) (Fig. 3c). Furthermore, correlating the loss of KAT6A::NCOA2 binding with changes in gene expression, we found that the loss of fusion protein binding was primarily associated with gene expression downregulation as early as 12 h (Fig. 3d).

To assess whether the observed changes in bulk gene expression induced by WM-1119 treatment were consistent across all cells, we conducted single-cell transcriptomic analysis throughout WM-1119 treatment on MT2 cells. These experiments revealed rapid, complete, sustained segregation of WM-1119-treated cells from DMSO control cells into distinct groups (Fig. 3e), and upregulation of markers of differentiation (*Cebpe*,* Itgam*,* CD68*) in WM-1119-treated cells compared to DMSO-treated cells (Fig. 3f). Similar gene sets to those observed in bulk RNA-Seq (Fig. 3c) related to myeloid development and genes downregulated by *HoxA9* and *Meis1* were enriched by GSEA for single cells (Supplementary Fig S5). Overall, these cellular and molecular findings revealed that WM-1119 treatment of KAT6Ar AML results in swift suppression of leukemia and stemness-associated genes and facilitates the robust and complete release of the myeloid differentiation block. Consequently, our data provide compelling evidence for the therapeutic potential of KAT6A inhibitors in treating KAT6Ar AMLs.

### Development of new KAT6A mouse models

KAT6A is a genetic vulnerability in KMT2A rearranged (KMT2Ar) AML [16–18], as corroborated by the DepMap portal (https://depmap.org/portal/) [19, 20]. Notably, members of the KMT2A complex, including ENL (MLLT1), KMT2A (MLL), DOT1L, and ASH1L, are among the top KAT6A co-dependencies in the CRISPR screen DepMap database [19]. These findings, alongside shared aberrant gene expression profiles [60, 61], suggest cooperation between KMT2A and KAT6A in sustaining KMT2Ar and KAT6Ar AMLs. While these results underscore the potential of targeting KAT6A as a therapeutic strategy for KMT2Ar AMLs, many investigations have primarily focused on either knocking down or knocking out the entire protein. Consequently, it remains unclear whether therapeutic interventions should specifically target KAT6A’s KAT activity or the protein as a whole and what adverse effects might accompany each approach. To address this crucial question, we utilized two new mouse models to delineate and compare the requirement for the complete KAT6A protein versus its KAT activity in KMT2Ar AMLs.

In the first model, we engineered a mutant variant allele of *Kat6a* (KAT6A MUT), disrupting the KAT catalytic domain through a double point mutation (Q654E/G657E, Supplementary Fig S7a). This mutation abolishes the residual KAT activity observed in vitro with the previous single mutation model (G657E) [11, 62]. We confirmed that similarly to this previous G657E model, mutations of both *Kat6a* alleles resulted in embryonic lethality. Apart from comprehensively inhibiting KAT activity, this model allows for a specific, and complete, inhibition of KAT6A’s KAT activity, circumventing the known impact of WM-1119 on the KAT activities of other MYST members (KAT7, KAT6B) [22, 63].

Additionally, we developed a *Kat6a* conditional knockout model (KAT6A^FL/FL^, Supplementary Fig S7b). In this model, Cre-induced deletion of exon 4 *of Kat6a* results in the creation of a premature stop codon and nonsense-mediated RNA decay *of Kat6a* transcripts. We first confirmed that constitutive deletion using PGK-Cre mirrors the embryonic lethality observed in previous total KAT6A knockout models (Supplementary Fig S7c). To explore the relevance of KAT6A in adult hematopoiesis, we crossed these KAT6A^FL/FL^ mice with *Vav-Cre* mice, where CRE expression primarily targets the hematopoietic system. Our results demonstrate that Vav-Cre/ KAT6A^FL/FL^ mice reach adulthood and remain healthy, despite efficient *Kat6a* deletion in bone marrow cells (Supplementary Fig S7d). Analysis of various hematopoietic compartments revealed primarily reduced B cells and phenotypic HSCs (Supplementary Fig S7e and S7f), alongside impaired bone marrow cell reconstitution in recipients (Supplementary Fig S7g). These observations suggest that although KAT6A is critical for B cell development and HSC maintenance in adult hematopoiesis, its deletion does not lead to increased morbidity or mortality. These results are consistent with a previous study [8] and indicate a potential therapeutic window for targeting KAT6A.

### Inhibiting the catalytic activity of KAT6A minimally affects KMT2A::MLLT3 AML growth

**Fig. 4:**
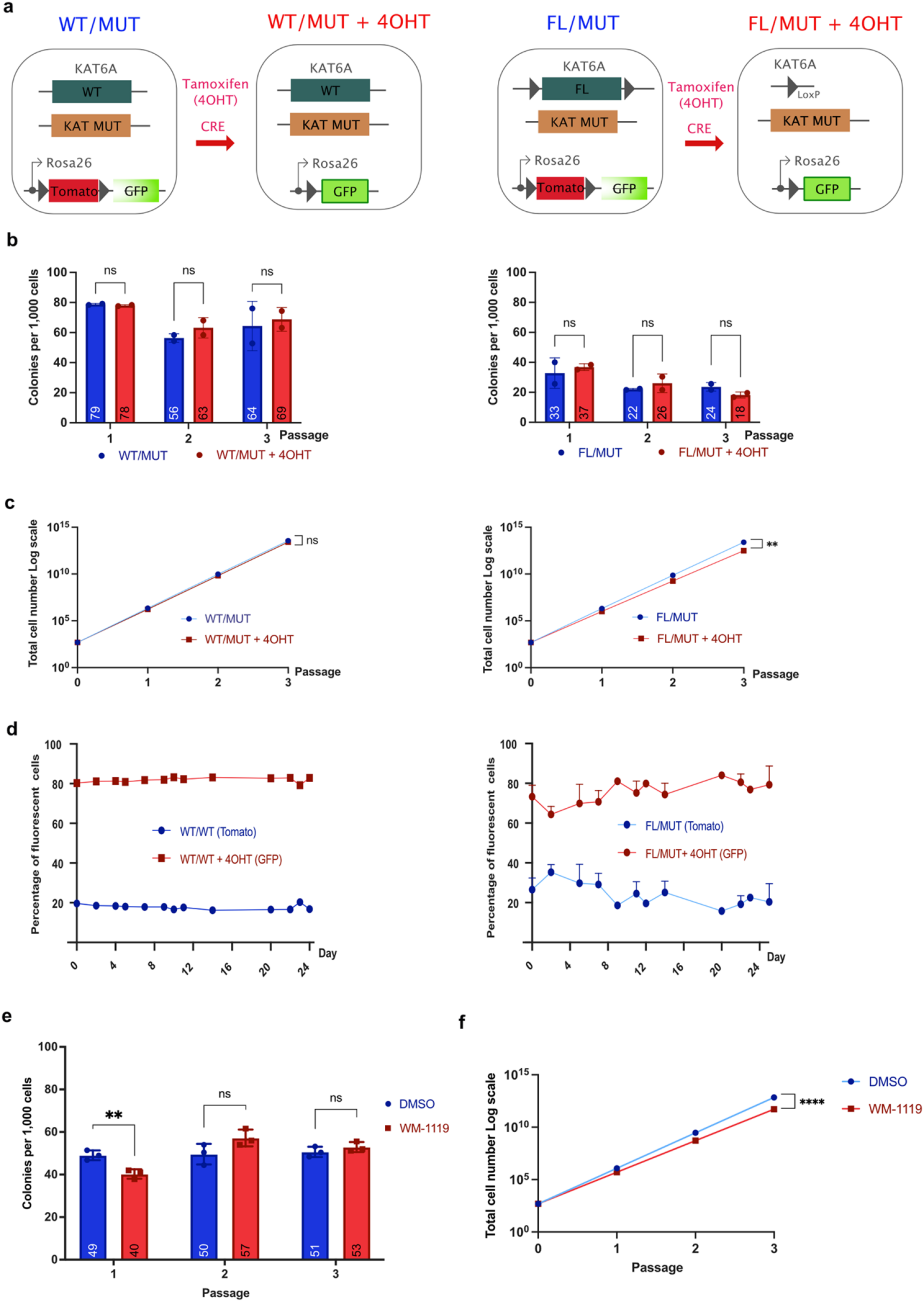
Inhibiting the catalytic activity of KAT6A minimally affects KMT2A::MLLT3 AML growth. **a**, Schematic showing the genotypic effects of tamoxifen inducible Cre-loxP. **b**, Colony numbers and **c**, total cells counted after plating of 1,000 cells at each MeC round for KMT2A::MLLT3 AML cell lines with genotypes as detailed with or without treatment with 4-hydroxytamoxifen (4OHT). (*n* = 2, error bars represent mean with standard deviation, two tailed unpaired t test, *p* ≥0.05 (ns), 0.01 to 0.05 (*), 0.001 to 0.01 (**), 0.0001 to 0.001 (***), < 0.0001 (****)). **d**, Liquid culture competition assays between cells undergoing Cre recombination following 4OHT treatment and those untreated for control cells (KAT6A^WT/WT^) and KAT mutant cells (KAT6A^FL/MUT^). **e**, Colony numbers counted and **f**, total cells counted for murine KMT2A::MLLT3 AML cells treated with DMSO or 1µM WM-1119 at each passage. (*n* = 3, two tailed unpaired t test, *p* ≥0.05 (ns), 0.01 to 0.05 (*), 0.001 to 0.01 (**), 0.0001 to 0.001 (***), < 0.0001 (****))

To assess the necessity of KAT activity, and circumvent the embryonic lethality observed in the absence of KAT6A KAT activity [7, 10], we combined the MUT allele with the conditional floxed functional *Kat6a* allele to generate KAT6A^FL/MUT^ adult mice. Additionally, we introduced a tamoxifen-inducible Cre-recombinase (CreERT2) [37] and a two-colour fluorescent CRE activity reporter allele mTmG (membrane Tomato / membrane GFP) [38]. 4-hydroxytamoxifen (4OHT) induction of recombination leads to the loss of the functional *Kat6a* allele, a switch from Tomato to GFP expression, and the exclusive expression of catalytically dead KAT6A (KAT6A^KO/MUT^, Fig. 4a). Murine KMT2A::MLLT3 AML cell lines were generated by retroviral transfection of cKIT^+^ cells from KAT6A^FL/MUT^ bone marrow. To account for the influence of CRE activity on AML cells, we also created KMT2A::MLLT3 AML lines from mice carrying a wild-type, instead of the floxed, KAT6A allele (KAT6A^WT/MUT^). These transformed cells were selected through multiple rounds of methylcellulose replating.

Initially, we assessed the requirement of KAT6A’s KAT activity in serial replating of KMT2A::MLLT3 cells. No statistically significant reduction in colony numbers was observed in KMT2A::MLLT3 AML cells where KAT6A’s KAT activity was abrogated compared to KAT6A^WT/MUT^, KAT6A^WT/WT^ and KAT6A^WT/FL^ controls (Fig. 4b and Supplementary Fig S8). However, a slight reduction in cumulative cell numbers was noted in this group (Fig. 4c). Subsequently, we set up cellular competition assays in liquid culture, in which KAT6A^FL/MUT^ cells, where KAT6A KAT is still functional (Tomato, KAT6A^FL/MUT^) were seeded at 20–25% of the total cell number with 75–80% of cells with abrogated KAT6A KAT activity (sorted GFP^+^ cells, KAT6A^FL/MUT + 4OHT^). Sequential FACS analyses over more than 3 weeks indicated that KMT2A::MLLT3 AML cells lacking KAT6A’s KAT activity were not outcompeted (Fig. 4d).

Finally, we pharmacologically targeted KAT6A’s KAT activity in murine KMT2A::MLLT3 AML with WM-1119. While there was initially a minor reduction in clonogenicity in the treated group, the loss of colony numbers was not sustained upon further replating (Fig. 4e). Although statistically significant, the difference in cellular proliferation between treated and untreated groups was relatively modest (Fig. 4f). Taken together, these findings indicate that genetically, or pharmacologically, targeting KAT6A’s KAT activity minimally affects the growth of KMT2A::MLLT3 AML cells.

### Deletion of KAT6A dramatically affects leukemic potential in murine KMT2A::MLLT3 AML

**Fig. 5:**
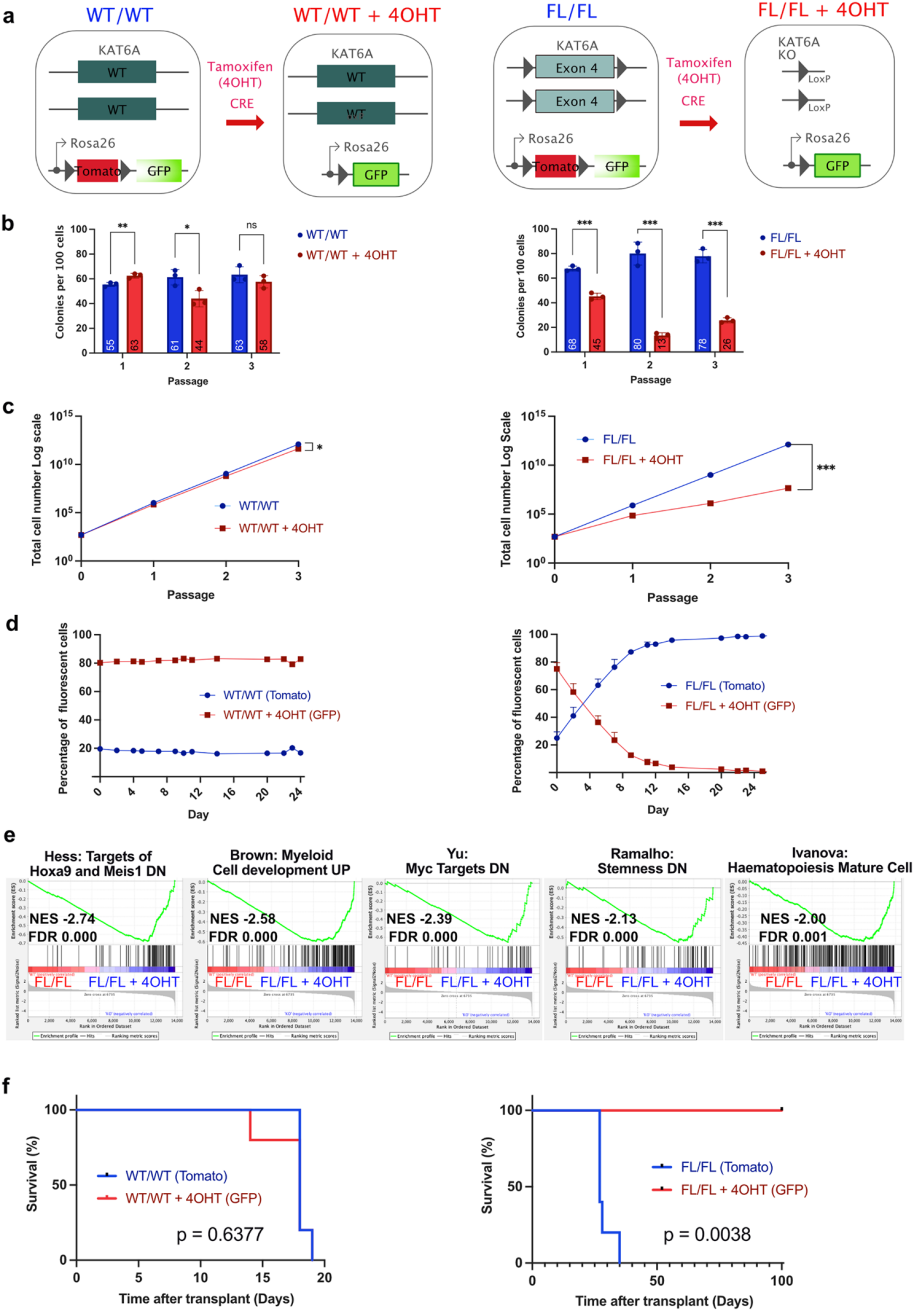
Deletion of Kat6a leads to loss of leukemic potential in KMT2A::MLLT3 AML cells. **a**, Schematic showing the genotypic effects of tamoxifen inducible Cre-loxP. **b**, Colonies counted and **c**, total cells counted after plating of 1000 cells at each MeC round for Kat6a^WT/WT^ or Kat6a^FL/FL^ KMT2A::MLLT3 AML cell lines with or without treatment with 4OHT. For 4OHT treated cells, cells plated with 50nM 4OHT in round 1 and 10nM 4OHT rounds 2 and 3. (*n* = 3, error bars represent mean with standard deviation, two tailed unpaired t test, *p* ≥0.05 (ns), 0.01 to 0.05 (*), 0.001 to 0.01 (**), 0.0001 to 0.001 (***), < 0.0001 (****)). **d**, Competition assays showing the percentage of 4OHT treated or untreated cells over time. **e**, Leading edge plots for selected gene sets enriched in murine KMT2A::MLLT3 *Kat6a* KO AML cells at 4 and 7 days post 4OHT addition. NES, normalised enrichment score, FDR, false discovery rate. **f**, Kaplan-Meier plots for primary KMT2A::MLLT3 AML transplantation experiment (*n* = 5 in each group). P value Log-rank (Mantel-Cox) test, *p* ≥0.05 (ns), 0.01 to 0.05 (*), 0.001 to 0.01 (**), 0.0001 to 0.001 (***), < 0.0001 (****))

To explore the requirement of the full KAT6A protein, we next generated murine KMT2A::MLLT3 AMLs with c-KIT^+^ cells from the bone marrow of KAT6A^FL/FL^ mice, which also carried the Cre^ERT2^ and mTmG alleles (Fig. 5a). To account for the potential influence of CRE activity on AML cells, we established KMT2A::MLLT3 AML lines from mice harbouring wild-type KAT6A alleles (KAT6A^WT/WT^), along with the tamoxifen-inducible Cre and the mTmG reporter. These transformed cells underwent selection through multiple rounds of methylcellulose replating.

**Fig. 6:**
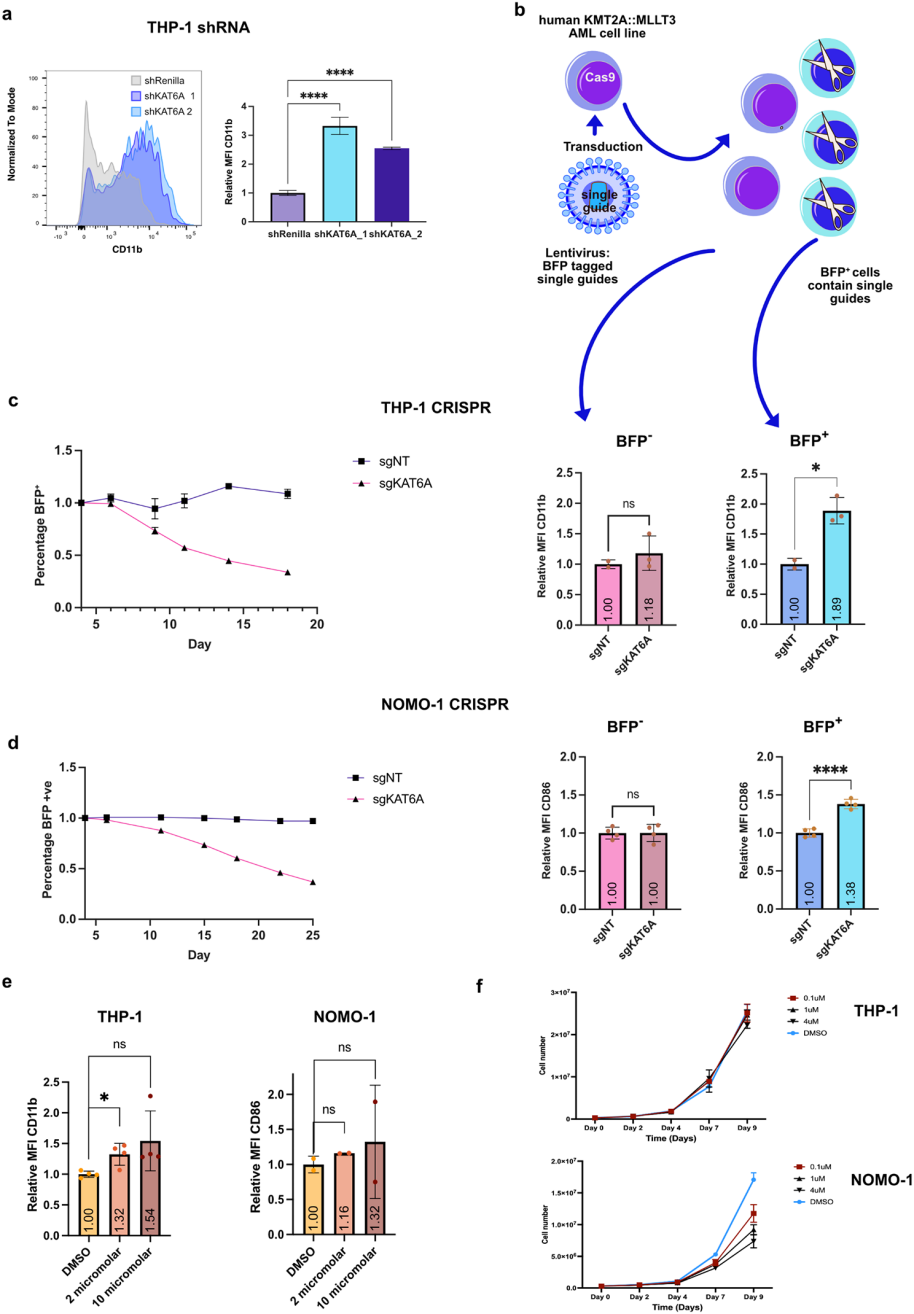
Deletion of KAT6A affects growth of human KMT2A::MLLT3 AML cell lines. **a**, Histogram showing marker of myeloid differentiation CD11b expression in THP-1 cells 6 days after induction of control shRNA against Renilla (shRenilla) or induction of shRNA’s targeting KAT6A (shKAT6A_1 and shKAT6A_2) and histogram showing relative mean fluorescence intensity of CD11b 3 days after induction of the same shRNAs. (*n* = 3, two tailed unpaired t test, *p* ≥0.05 (ns), 0.01 to 0.05 (*), 0.001 to 0.01 (**), 0.0001 to 0.001 (***), < 0.0001 (****)). **b**, Schematic showing single guides expressing BFP and targeting either KAT6A (sgKAT6A_1) or with no target (sgNT) were lentivirally transduced in to THP-1 or NOMO-1 cells. CRISPR editing occurs in BFP positive cells only. **c**, THP-1 and **d**, NOMO-1 CRISPR mediated cellular competition assay. BFP expressing cells are recorded relative to BFP expression four days after transduction over 23 or 25 days (*n* = 2). Relative mean fluorescence intensity (MFI) of the myeloid differentiation markers CD11b or CD86 was recorded at day 4 for cells expressing BFP in which CRISPR editing occurred or BFP negative cells in which there was no CRISPR editing (*n* = 3, two tailed unpaired t test, *p* ≥0.05 (ns), 0.01 to 0.05 (*), 0.001 to 0.01 (**), 0.0001 to 0.001 (***), < 0.0001 (****)). **e**, Relative mean fluorescence intensity (MFI) of the myeloid differentiation markers CD11b or CD86 4 days after treatment with the stated doses of WM-1119 at day 0 in THP-1 and NOMO-1 cells (*n* = 4 THP-1, *n* = 2 NOMO-1, both two tailed unpaired t test, *p* ≥0.05 (ns), 0.01 to 0.05 (*), 0.001 to 0.01 (**), 0.0001 to 0.001 (***), < 0.0001 (****)). **f**, Cell counts for THP-1 and NOMO-1 cells treated with the stated concentrations of WM-1119 every two days (*n* = 3). All error bars represent mean with standard deviation

Initially, we assessed the requirement of KAT6A in the serial replating of KMT2A::MLLT3 cells. The addition of 4OHT in the methylcellulose cultures resulted in a significant reduction of clonogenicity (Fig. 5b) and a substantial reduction in cumulative cell number (Figs. 5c and 6 logs after 3 replatings) in tamoxifen treated KAT6A^FL/FL^ cells (KAT6A^FL/FL+4OHT^). We then conducted cellular competition assays, wherein KMT2A::MLLT3 AML cells treated with 4OHT and FACS sorted for GFP expression were seeded at 75–80% of the total cells alongside 20–25% of untreated cells. While WT cells treated with 4OHT, in which *Kat6a* was not deleted (KAT6A^WT/WT+4OHT^), continued to proliferate, cells in which *Kat6a* was deleted (KAT6A^FL/FL+4OHT^) were rapidly outcompeted by untreated cells (Fig. 5d). GSEA of bulk RNA of KMT2A::MLLT3 KAT6A^FL/FL^ and KAT6A^FL/FL+4OHT^ AML cells indicated that the deletion of *Kat6a* was associated with the loss of normal hematopoietic stemness, reduction in HOXA9, MEIS1, KMT2A, and MYC target programs, and increased myeloid development (Fig. 5e). Collectively, these findings underscore the significance of KAT6A for the proliferation and replating of KMT2A::MLLT3 AML cells in vitro, along with its role in maintaining leukemic programs and blocking myeloid differentiation.

We then investigated if *Kat6a* deletion impaired AML in vivo. For this, c-KIT^+^ cells from different KAT6A GEMMs were transduced with KMT2A::MLLT3 encoding retrovirus and injected into irradiated mice to generate primary AMLs. The resulting primary KMT2A::MLLT3 AML cells underwent brief ex vivo treatment with 4OHT, followed by FACS sorting. GFP and Tomato cells were then injected into sub-lethally irradiated mice, and overall survival (OS) was monitored. Tamoxifen treatment alone had no effect on mouse survival, with animals injected with KAT6A^WT/WT^ KMT2A::MLLT3 AML dying rapidly irrespective of exposure to 4OHT (Fig. 5f left). In contrast, deletion of *Kat6a* using 4OHT significantly improved OS. None of these mice died within 100 days, whereas all those injected with the same KMT2A::MLLT3 AML but retaining KAT6A expression were deceased within 35 days (Fig. 5f right).

In summary, these experiments highlight the critical role of Kat6a in the proliferation of KMT2A::MLLT3 AML in vitro and demonstrate its significant functional impact on leukemogenesis in vivo when deleted.

### Deletion of KAT6A affects growth of human KMT2A::MLLT3 AML cell lines

We next aimed to validate these findings in human AML cells. Initially, we implemented an inducible shRNA approach with two shRNAs targeting *KAT6A* along with a control shRNA against Renilla in THP-1 KMT2A::MLLT3 cells. After 3 days of doxycycline induction of the shRNA, we observed approximately 75% knockdown of KAT6A mRNA (Supplementary Fig S9a) and a notable increase in myeloid differentiation, as evidenced by elevated CD11b expression from day 3 onwards (Fig. 6a and Supplementary Fig S9b). These results were then further corroborated through CRISPR-mediated KAT6A knockout. We transduced human AML KMT2A::MLLT3 cell lines THP-1 and NOMO1 constitutively expressing Cas9 with viruses encoding BFP and either a non-targeting guide (sgNT) or a guide against KAT6A (sgKAT6A) (Fig. 6b, Supplementary Fig S10). BFP^+^ cells carrying the guide against KAT6A (sgKAT6A) were depleted upon culture of both cell lines, contrasting with cells carrying the non-targeting guide (sgNT) (Fig. 6c and d). Furthermore, a significant increase in the myeloid markers CD11b or CD86 was observed in cells carrying sgKAT6A (BFP^+^) compared to cells not successfully transduced (BFP^−^) (Fig. 6c and d). Even if CRISPR-mediated KAT6A knockout is not total (Supplementary Fig S10), treatment of THP-1 and NOMO-1 cells with WM-11,119 resulted in less significant induction of myeloid differentiation (Fig. 6e) with hardly any effect on proliferation in the case of THP-1 and some decrease in proliferation for NOMO-1 (Fig. 6f). Altogether, these findings indicate that akin to murine AML KMT2Ar cells, human AML KMT2A::MLLT3 cells exhibit enhanced myeloid differentiation and reduced proliferation in response to decreased KAT6A levels. Weaker myeloid differentiation is observed upon targeting KAT6A’s KAT activity with WM-1119.

## Discussion

The recurrent translocation of KAT6A in AML prompted our exploration of the therapeutic potential of targeting it using newly available inhibitors. We demonstrated here that even small doses of WM-1119 effectively suppress the *in vitr*o growth of a murine KAT6Ar AML cell line (MT2*)*, abolishing clonogenic potential, arresting cell cycle progression, downregulating the expression of leukemia related and stemness-associated genes, whilst promoting the upregulation of myeloid differentiation. Similar reductions in proliferation were observed in vitro with primary human AML cells carrying the KAT6A::CREBBP translocation. Mechanistically, this effect is associated with the loss of binding of the fusion protein KAT6A::NCOA2 to key leukemia genes. Nevertheless, we cannot discount additional effects of WM-1119 inhibition, as our initial analyses indicate that acetylation of numerous proteins, often beyond histones, is influenced by the treatment (Supplementary Fig S11, Supplementary Table 3). In addition, the higher KAT6A::NCOA2 ChIP signals observed at conserved peaks in the presence of WM-1119 (Supplementary Fig. S2d) suggest that this compound may also stabilize the KAT6A fusion protein or increase the accessibility of the Ty1 tag. The effectiveness of the treatment seems to depend on targeting both the KAT6A::NCOA2 fusion protein and the endogenous KAT6A protein. Indeed, evaluation of the genetic loss of the KAT activity of the endogenous KAT6A in MT2 cells engineered with KAT6A^MUT/FL^ cells showed significantly less impact on growth compared to treatment with WM-1119, which targets both the fusion and endogenous proteins (data not shown). Similarly, deletion of the endogenous KAT6A protein in MT2 cells derived from KAT6A^FL/FL^ cells resulted in significantly less affected growth compared to WM-1119 treatment (data not shown).

Given that over 60% of patients with KAT6A::CREBBP translocated AML, even after undergoing allogeneic transplantation (currently the most effective treatment), still succumb within 5 years [23], there is a pressing need for additional therapeutic strategies. Our findings offer evidence supporting the potential effectiveness of KAT6A inhibition, potentially laying the groundwork for informed trials of these medications in individual patients. In this regard, ongoing early-phase trials of KAT6A inhibitors in breast, lung, and prostate cancer patients (NCT04606446, trial registered October 28 2020) will provide crucial insights into the presence or absence of adverse safety signals, guiding future potential clinical applications in AML management.

Multiple observations suggest that KAT6A and the histone methyltransferase KMT2A (MLL) co-regulate similar mechanisms. Firstly, KMT2A and KAT6A have been shown to interact and jointly regulate HOXA gene expression in human cord blood cells during normal early hematopoiesis [14]. Secondly, KAT6Ar AML and KMT2Ar AML exhibit similar gene expression profiles [60, 61], and KAT6A is now recognized as a genetic vulnerability in KMT2Ar AML, with KMT2A being one of its top co-dependencies [17, 19]. In line with this, Yan et al. [17] demonstrated that pharmacological inhibition of KAT6A inhibits KMT2A::MLLT3 growth, and our own research has partially replicated these findings. Consequently, KAT6A inhibitors may prove effective in combination treatments for KMT2A::MLLT3 AML. Indeed, a recent CRISPR screen in cells treated with a menin inhibitor [64] using the same KMT2A::MLLT3 cell line as [17] identified KAT6A as the top co-dependency. In vitro assays confirmed synergistic loss of viability upon combination of WM-1119 with menin inhibition (SND-50469) in a KMT2A::MLLT3 cell line and a KMT2A::AFF4 cell line [64]. Further investigation in other AML subtypes, including other KMT2A translocations with distinct prognoses and biology compared to KMT2A::MLLT3 [65, 66], is warranted.

KAT6A is an attractive target due to the apparent therapeutic window revealed in our study. Furthermore, whilst knockout of KAT6A results in embryonic lethality [9], conditional knockout of KAT6A in the adult hematopoietic system is well-tolerated, maintaining relatively normal hematopoiesis [8, 10]. Moreover, individuals with KAT6A syndrome, even those harbouring nonsense mutations, typically exhibit normal hematopoiesis alongside the syndromic hallmark of neurodevelopmental disability [67]. To fully exploit this promising therapeutic window, the development of highly specific KAT6A-targeted treatments is imperative to minimize potential side effects. It is crucial to acknowledge that WM-1119, while effective in inducing myeloid differentiation (Yan et al. [17], Fig. 6e and Supplementary Fig S12) and having some none-uniform effect on proliferation (Fig. 6f), lacks complete specificity as it also inhibits KAT6B and KAT7 [22, 63]. To model the consequences of highly specific KAT6A KAT inhibition in KMT2Ar AMLs and circumvent potential confounding effects of inhibiting other MYST family members, we adopted a genetic approach. While total KAT inhibition showed a negative impact on leukemic transcriptomic programs in this murine model of KMT2A::MLLT3 AML, the observed phenotypes were less pronounced than when the entire KAT6A protein was removed. These findings suggest that additional functions of KAT6A might play critical roles in maintaining KMT2Ar AMLs. Indeed, KAT6A functions as a co-activator of hematopoietic-specific transcription factors RUNX1 and PU.1 and operates as a tetramer with BRPF1/2/3, ING5, and EAF6. Moreover, KAT6A and KMT2A interact within large complexes at promoters (Supplementary Fig S2), potentially rendering these complexes more sensitive to the loss of the entire KAT6A protein and its scaffolding role than to mere inhibition of its catalytic activity. We anticipate that genetic knockout effects may be therapeutically mimicked by leveraging targeted protein degradation through proteolysis-targeting chimeric molecules (PROTACs). PROTACs are heterobifunctional small molecules wherein a ligand binding an E3 ligase is covalently linked to a ligand binding the target protein, facilitating its ubiquitylation and subsequent proteasomal degradation [68]. This catalytic approach enables a single molecule to mediate the degradation of multiple targeted proteins [68], thereby abolishing all targeted protein functions, including enzymatic and scaffolding roles. The availability of KAT6A ligands, such as WM-1119, may serve as a starting point for developing PROTACs against KAT6A.

In summary, our study highlights KAT6A as a genetic dependency in KAT6Ar and KMT2Ar AML, making it a promising therapeutic target. In particular, our study reveals a dramatic therapeutic efficacy of currently available KAT6A inhibitors in KAT6Ar AMLs. This efficacy stems from their ability to directly target the KAT activity of the oncogenic driver fusion protein sustaining the leukemia. Conversely, the catalytic activity of KAT6A plays a lesser role in KMT2Ar leukemogenicity. Our findings suggest that targeting the entire KAT6A protein may represent a more effective therapeutic strategy. Exploring the potential of targeted KAT6A degradation in KMT2Ar AML holds particular promise.

## Electronic supplementary material

Below is the link to the electronic supplementary material.

Supplementary Material 1

Supplementary Material 2

Supplementary Material 3

Supplementary Material 4

Supplementary Material 5

Supplementary Material 6

Supplementary Material 7

Supplementary Material 8

Supplementary Material 9

Supplementary Material 10

Supplementary Material 11

## Data Availability

Raw sequencing files and processed data are available in GEO (PRJNA1100482).

## References

[1] Shlush LI, Zandi S, Mitchell A, Chen WC, Brandwein JM, Gupta V, et al. Identification of pre-leukaemic haematopoietic stem cells in acute leukaemia. Nature. 2014;506:328–33. 24522528 10.1038/nature13038 PMC4991939

[2] Abelson S, Collord G, Ng SWK, Weissbrod O, Mendelson Cohen N, Niemeyer E, et al. Prediction of acute myeloid leukaemia risk in healthy individuals. Nature. 2018;559:400–4. 29988082 10.1038/s41586-018-0317-6 PMC6485381

[3] Chopra M, Bohlander SK. The cell of origin and the leukemia stem cell in acute myeloid leukemia. Genes Chromosomes Cancer. 2019;58:850–8. 31471945 10.1002/gcc.22805

[4] Kantarjian H, Kadia T, DiNardo C, Daver N, Borthakur G, Jabbour E, et al. Acute myeloid leukemia: current progress and future directions. Blood Cancer J. 2021;11:41. 33619261 10.1038/s41408-021-00425-3 PMC7900255

[5] Yang XJ, Ullah M. MOZ and MORF, two large MYSTic HATs in normal and cancer stem cells. Oncogene. 2007;26:5408–19. 17694082 10.1038/sj.onc.1210609

[6] Perez-Campo FM, Costa G, Lie-a-Ling M, Kouskoff V, Lacaud G. The MYSTerious MOZ, a histone acetyltransferase with a key role in haematopoiesis. Immunology. 2013;139:161–5. 23347099 10.1111/imm.12072 PMC3647182

[7] Thomas T, Corcoran LM, Gugasyan R, Dixon MP, Brodnicki T, Nutt SL, et al. Monocytic leukemia zinc finger protein is essential for the development of long-term reconstituting hematopoietic stem cells. Genes Dev. 2006;20:1175–86. 16651658 10.1101/gad.1382606 PMC1472476

[8] Sheikh BN, Yang Y, Schreuder J, Nilsson SK, Bilardi R, Carotta S, et al. MOZ (KAT6A) is essential for the maintenance of classically defined adult hematopoietic stem cells. Blood. 2016;128:2307–18. 27663673 10.1182/blood-2015-10-676072

[9] Katsumoto T, Aikawa Y, Iwama A, Ueda S, Ichikawa H, Ochiya T, et al. MOZ is essential for maintenance of hematopoietic stem cells. Genes Dev. 2006;20:1321–30. 16702405 10.1101/gad.1393106 PMC1472906

[10] Perez-Campo FM, Borrow J, Kouskoff V, Lacaud G. The histone acetyl transferase activity of monocytic leukemia zinc finger is critical for the proliferation of hematopoietic precursors. Blood. 2009;113:4866–74. 19264921 10.1182/blood-2008-04-152017 PMC2686138

[11] Perez-Campo FM, Costa G, Lie-a-Ling M, Stifani S, Kouskoff V, Lacaud G. MOZ-Mediated repression of p16INK4a is critical for the Self-Renewal of neural and hematopoietic stem cells. Stem Cells. 2014;32:1591–601. 24307508 10.1002/stem.1606 PMC4237135

[12] Kitabayashi I, Aikawa Y, Nguyen LA, Yokoyama A, Ohki M. Activation of AML1-mediated transcription by MOZ and inhibition by the MOZ-CBP fusion protein. EMBO J. 2001;20:7184–96. 11742995 10.1093/emboj/20.24.7184 PMC125775

[13] Yang XJ. MOZ and MORF acetyltransferases: molecular interaction, animal development and human disease. Biochim Biophys Acta Mol Cell Res. 2015;1853:1818–26. 10.1016/j.bbamcr.2015.04.014 25920810

[14] Paggetti J, Largeot A, Aucagne R, Jacquel A, Lagrange B, Yang XJ, et al. Crosstalk between leukemia-associated proteins MOZ and MLL regulates HOX gene expression in human cord blood CD34 cells. Oncogene. 2010;29:5019–31. 20581860 10.1038/onc.2010.254

[15] Jung N, Dai B, Gentles AJ, Majeti R, Feinberg AP. An LSC epigenetic signature is largely mutation independent and implicates the HOXA cluster in AML pathogenesis. Nat Commun. 2015;6. 10.1038/ncomms9489 PMC4633733 26444494

[16] Au YZ, Gu M, De Braekeleer E, Gozdecka M, Aspris D, Tarumoto Y, et al. KAT7 is a genetic vulnerability of acute myeloid leukemias driven by MLL rearrangements. Leukemia. 2020. 10.1038/s41375-020-1001-z. 32764680 10.1038/s41375-020-1001-z PMC7610570

[17] Yan F, Li J, Milosevic J, Petroni R, Liu S, Shi Z, et al. KAT6A and ENL Form an Epigenetic Transcriptional Control Module to drive critical leukemogenic gene-expression programs. Cancer Discov. 2022;12:792–811. 34853079 10.1158/2159-8290.CD-20-1459 PMC8916037

[18] Katsumoto T, Ogawara Y, Yamagata K, Aikawa Y, Goitsuka R, Nakamura T, et al. MOZ is critical for the development of MOZ/MLL fusion–induced leukemia through regulation of Hoxa9/Meis1 expression. Blood Adv. 2022;6:5527–37. 35947126 10.1182/bloodadvances.2020003490 PMC9577624

[19] DepMap B. DepMap 20Q2 Public. figshare. Dataset. 2020;:10.6084/m9.figshare.12280541.v4

[20] Meyers RM, Bryan JG, McFarland JM, Weir BA, Sizemore AE, Xu H, et al. Computational correction of copy number effect improves specificity of CRISPR-Cas9 essentiality screens in cancer cells. Nat Genet. 2017;49:1779–84. 29083409 10.1038/ng.3984 PMC5709193

[21] Dempster J, Rossen J, Kazachkova M, Pan J, Kugener G, Root D et al. Extracting Biological insights from the Project Achilles Genome-Scale CRISPR screens in Cancer Cell lines. BioRXiv. 2019. 10.1101/720243

[22] Baell JB, Leaver DJ, Hermans SJ, Kelly GL, Brennan MS, Downer NL, et al. Inhibitors of histone acetyltransferases KAT6A/B induce senescence and arrest tumour growth. Nature. 2018;560:253–7. 30069049 10.1038/s41586-018-0387-5

[23] Kayser S, Hills RK, Langova R, Kramer M, Guijarro F, Sustkova Z, et al. Characteristics and outcome of patients with acute myeloid leukaemia and t(8;16)(p11;p13): results from an International Collaborative Study*. Br J Haematol. 2021;192:832–42. 33529373 10.1111/bjh.17336

[24] Rozman M, Camós M, Colomer D, Villamor N, Esteve J, Costa D, et al. Type I MOZ/CBP (MYST3/CREBBP) is the most common chimeric transcript in Acute myeloid leukemia with t(8;16)(p11;p13) translocation. Genes Chromosomes Cancer. 2004;40:140–5. 15101047 10.1002/gcc.20022

[25] Wong KF, Yuen HL, Siu LLP, Pang A, Kwong YL. T(8;16)(P11;P13) predisposes to a transient but potentially recurring neonatal leukemia. Hum Pathol. 2008;39:1702–7. 18657848 10.1016/j.humpath.2008.02.018

[26] Brown T, Swansbury J, Taj MM. Prognosis of patients with t(8;16)(p11;p13) acute myeloid leukemia. Leuk Lymphoma. 2012;53:338–41. 21846182 10.3109/10428194.2011.614703

[27] Schmidt HH, Strehl S, Thaler D, Strunk D, Sill H, Linkesh W, et al. RT-PCR and FISH analysis of acute myeloid leukemia with t(8;16)(p11;p13) and chimeric MOZ and CBP transcripts: breakpoint cluster region and clinical implications. Leukemia. 2004;18:1115–21. 15085163 10.1038/sj.leu.2403353

[28] Crowley JA, Wang Y, Rapoport AP, Ning Y. Detection of MOZ-CBP fusion in acute myeloid leukemia with 8;16 translocation [8]. Leukemia. 2005;19:2344–5. 16193081 10.1038/sj.leu.2403971

[29] Kitabayashi I, Aikawa Y, Yokoyama A, Hosoda F, Nagai M, Kakazu N, et al. Fusion of MOZ and p300 histone acetyltransferases in acute monocytic leukemia with a t(8;22)(p11;q13) chromosome translocation. Leukemia. 2001;15:89–94. 11243405 10.1038/sj.leu.2401983

[30] Chaffanet M, Gressin L, Preudhomme C, Soenen-Cornu V, Birnbaum D, Pébusque MJ. MOZ is fused to p300 in an acute monocytic leukemia with t(8;22). Genes Chromosomes Cancer. 2000;28:138–44. 10824998 10.1002/(sici)1098-2264(200006)28:2<138::aid-gcc2>3.0.co;2-2

[31] Carapeti M, Aguiar RCT, Goldman JM, Cross NCP. A novel fusion between MOZ and the nuclear receptor coactivator TIF2 in acute myeloid leukemia. Blood. 1998;91:3127–33. 9558366

[32] Carapeti M, Aguiar RCT, Watmore AE, Goldman JM, Cross NCP. Consistent fusion of MOZ and TIF2 in AML with inv(8)(p11q13). Cancer Genet Cytogenet. 1999;113:70–2. 10459350 10.1016/s0165-4608(99)00007-2

[33] Liang J, Prouty L, Williams BJ, Dayton MA, Blanchard KL. Acute mixed lineage leukemia with an inv(8)(p11q13) resulting in fusion of the genes for MOZ and TIF2. Blood. 1998;92:2118–22. 9731070

[34] Klein BJ, Lalonde M-E, Côté J, Yang X-J, Kutateladze TG. Crosstalk between epigenetic readers regulates the MOZ/MORF HAT complexes. Epigenetics. 2014;9:186–93. 24169304 10.4161/epi.26792 PMC3962528

[35] Meyer C, Burmeister T, Gröger D, Tsaur G, Fechina L, Renneville A, et al. The MLL recombinome of acute leukemias in 2017. Leukemia. 2018;32:273–84. 28701730 10.1038/leu.2017.213 PMC5808070

[36] Hodgkins A, Farne A, Perera S, Grego T, Parry-Smith DJ, Skarnes WC, et al. WGE: a CRISPR database for genome engineering. Bioinformatics. 2015;31:3078–80. 25979474 10.1093/bioinformatics/btv308 PMC4565030

[37] Ventura A, Kirsch DG, McLaughlin ME, Tuveson DA, Grimm J, Lintault L, et al. Restoration of p53 function leads to tumour regression in vivo. Nature. 2007;445:661–5. 17251932 10.1038/nature05541

[38] Muzumdar MD, Tasic B, Miyamichi K, Li L, Luo L. A global double-fluorescent Cre reporter mouse. Genesis. 2007;45:593–605. 17868096 10.1002/dvg.20335

[39] Pelossof R, Fairchild L, Huang C-H, Widmer C, Sreedharan VT, Sinha N, et al. Prediction of potent shRNAs with a sequential classification algorithm. Nat Biotechnol. 2017;35:350–3. 28263295 10.1038/nbt.3807 PMC5416823

[40] Dobin A, Davis CA, Schlesinger F, Drenkow J, Zaleski C, Jha S, et al. STAR: ultrafast universal RNA-seq aligner. Bioinformatics. 2013;29:15–21. 23104886 10.1093/bioinformatics/bts635 PMC3530905

[41] Liao Y, Smyth GK, Shi W. The R package rsubread is easier, faster, cheaper and better for alignment and quantification of RNA sequencing reads. Nucleic Acids Res. 2019;47:e47–47. 30783653 10.1093/nar/gkz114 PMC6486549

[42] Love MI, Huber W, Anders S. Moderated estimation of Fold change and dispersion for RNA-seq data with DESeq2. Genome Biol. 2014;15:550. 25516281 10.1186/s13059-014-0550-8 PMC4302049

[43] Stuart T, Butler A, Hoffman P, Hafemeister C, Papalexi E, Mauck WM, et al. Comprehensive Integration of Single-Cell Data. Cell. 2019;177:1888–e190221. 31178118 10.1016/j.cell.2019.05.031 PMC6687398

[44] Langmead B, Salzberg SL. Fast gapped-read alignment with Bowtie 2. Nat Methods. 2012;9:357–9. 22388286 10.1038/nmeth.1923 PMC3322381

[45] https://github.com/broadinstitute/picard.:https://github.com/broadinstitute/picard

[46] Amemiya HM, Kundaje A, Boyle AP. The ENCODE Blacklist: identification of problematic regions of the genome. Sci Rep. 2019;9:9354. 31249361 10.1038/s41598-019-45839-z PMC6597582

[47] Zhang Y, Liu T, Meyer CA, Eeckhoute J, Johnson DS, Bernstein BE, et al. Model-based analysis of ChIP-Seq (MACS). Genome Biol. 2008;9:R137. 18798982 10.1186/gb-2008-9-9-r137 PMC2592715

[48] Lawrence M, Huber W, Pagès H, Aboyoun P, Carlson M, Gentleman R, et al. Software for Computing and Annotating genomic ranges. PLoS Comput Biol. 2013;9:e1003118. 23950696 10.1371/journal.pcbi.1003118 PMC3738458

[49] Ross-Innes CS, Stark R, Teschendorff AE, Holmes KA, Ali HR, Dunning MJ, et al. Differential oestrogen receptor binding is associated with clinical outcome in breast cancer. Nature. 2012;481:389–93. 22217937 10.1038/nature10730 PMC3272464

[50] Subramanian A, Tamayo P, Mootha VK, Mukherjee S, Ebert BL, Gillette MA, et al. Gene set enrichment analysis: a knowledge-based approach for interpreting genome-wide expression profiles. Proc Natl Acad Sci U S A. 2005;102:15545–50. 16199517 10.1073/pnas.0506580102 PMC1239896

[51] Weber LM, Jia Y, Stielow B, Gisselbrecht SS, Cao Y, Ren Y, et al. The histone acetyltransferase KAT6A is recruited to unmethylated CpG islands via a DNA binding winged helix domain. Nucleic Acids Res. 2023;51:574–94. 36537216 10.1093/nar/gkac1188 PMC9881136

[52] Becht DC, Klein BJ, Kanai A, Jang SM, Cox KL, Zhou B-R, et al. MORF and MOZ acetyltransferases target unmethylated CpG islands through the winged helix domain. Nat Commun. 2023;14:697. 36754959 10.1038/s41467-023-36368-5 PMC9908889

[53] Fan H, Lu J, Guo Y, Li D, Zhang ZM, Tsai YH, et al. BAHCC1 binds H3K27me3 via a conserved BAH module to mediate gene silencing and oncogenesis. Nat Genet. 2020;52:1384–96. 33139953 10.1038/s41588-020-00729-3 PMC8330957

[54] Collins CT, Hess JL. Deregulation of the HOXA9/MEIS1 axis in acute leukemia. Curr Opin Hematol. 2016;23:354–61. 27258906 10.1097/MOH.0000000000000245 PMC5653247

[55] Stavropoulou V, Kaspar S, Brault L, Sanders MA, Juge S, Morettini S, et al. MLL-AF9 expression in hematopoietic stem cells drives a highly invasive AML expressing EMT-Related genes linked to poor outcome. Cancer Cell. 2016;30:43–58. 27344946 10.1016/j.ccell.2016.05.011

[56] Vervoort SJ, van Boxtel R, Coffer PJ. The role of SRY-related HMG box transcription factor 4 (SOX4) in tumorigenesis and metastasis: friend or foe? Oncogene. 2013;32:3397–409. 23246969 10.1038/onc.2012.506

[57] Chen EY, Tan CM, Kou Y, Duan Q, Wang Z, Meirelles GV, et al. Enrichr: interactive and collaborative HTML5 gene list enrichment analysis tool. BMC Bioinformatics. 2013;14:128. 23586463 10.1186/1471-2105-14-128 PMC3637064

[58] Kuleshov MV, Jones MR, Rouillard AD, Fernandez NF, Duan Q, Wang Z, et al. Enrichr: a comprehensive gene set enrichment analysis web server 2016 update. Nucleic Acids Res. 2016;44:W90–7. 27141961 10.1093/nar/gkw377 PMC4987924

[59] Xie Z, Bailey A, Kuleshov MV, Clarke DJB, Evangelista JE, Jenkins SL, et al. Gene Set Knowledge Discovery with Enrichr. Curr Protoc. 2021;1:e90. 33780170 10.1002/cpz1.90 PMC8152575

[60] Haferlach T, Kohlmann A, Klein HU, Ruckert C, Dugas M, Williams PM, et al. AML with translocation t(8;16)(p11;p13) demonstrates unique cytomorphological, cytogenetic, molecular and prognostic features. Leukemia. 2009;23:934–43. 19194466 10.1038/leu.2008.388

[61] Camós M, Esteve J, Jares P, Colomer D, Rozman M, Villamor N, et al. Gene expression profiling of acute myeloid leukemia with translocation t(8;16)(p11;p13) and MYST3-CREBBP rearrangement reveals a distinctive signature with a specific pattern of HOX gene expression. Cancer Res. 2006;66:6947–54. 16849538 10.1158/0008-5472.CAN-05-4601

[62] Shima H, Yamagata K, Aikawa Y, Shino M, Koseki H, Shimada H, et al. Bromodomain-PHD finger protein 1 is critical for leukemogenesis associated with MOZ-TIF2 fusion. Int J Hematol. 2014;99:21–31. 24258712 10.1007/s12185-013-1466-x

[63] MacPherson L, Anokye J, Yeung MM, Lam EYN, Chan YC, Weng CF, et al. HBO1 is required for the maintenance of leukaemia stem cells. Nature. 2020;577:266–70. 31827282 10.1038/s41586-019-1835-6

[64] Fiskus W, Mill CP, Birdwell C, Davis JA, Das K, Boettcher S, et al. Targeting of epigenetic co-dependencies enhances anti-AML efficacy of Menin inhibitor in AML with MLL1-r or mutant NPM1. Blood Cancer J. 2023;13:1–13. 37055414 10.1038/s41408-023-00826-6 PMC10102188

[65] Grimwade D, Hills RK, Moorman AV, Walker H, Chatters S, Goldstone AH, et al. Refinement of cytogenetic classification in acute myeloid leukemia: determination of prognostic significance of rare recurring chromosomal abnormalities among 5876 younger adult patients treated in the United Kingdom Medical Research Council trials. Blood. 2010;116:354–65. 20385793 10.1182/blood-2009-11-254441

[66] Döhner H, Wei AH, Appelbaum FR, Craddock C, DiNardo CD, Dombret H, et al. Diagnosis and management of AML in adults: 2022 recommendations from an international expert panel on behalf of the ELN. Blood. 2022;140:1345–77. 35797463 10.1182/blood.2022016867

[67] Kennedy J, Goudie D, Blair E, Chandler K, Joss S, McKay V, et al. KAT6A syndrome: genotype–phenotype correlation in 76 patients with pathogenic KAT6A variants. Genet Sci. 2019;21:850–60. 10.1038/s41436-018-0259-2 PMC6634310 30245513

[68] Békés M, Langley DR, Crews CM. PROTAC targeted protein degraders: the past is prologue. Nat Rev Drug Discov. 2022;21:181–200. 35042991 10.1038/s41573-021-00371-6 PMC8765495

