# Supplementary figures and images for: The small inhibitor WM-1119 effectively targets KAT6A-rearranged AML, but not KMT2A-rearranged AML, despite shared KAT6 genetic dependency
Source: J Hematol Oncol. 2024 Oct 8;17:91. doi: 10.1186/s13045-024-01610-0 (PMC11462755; doi:10.1186/s13045-024-01610-0)

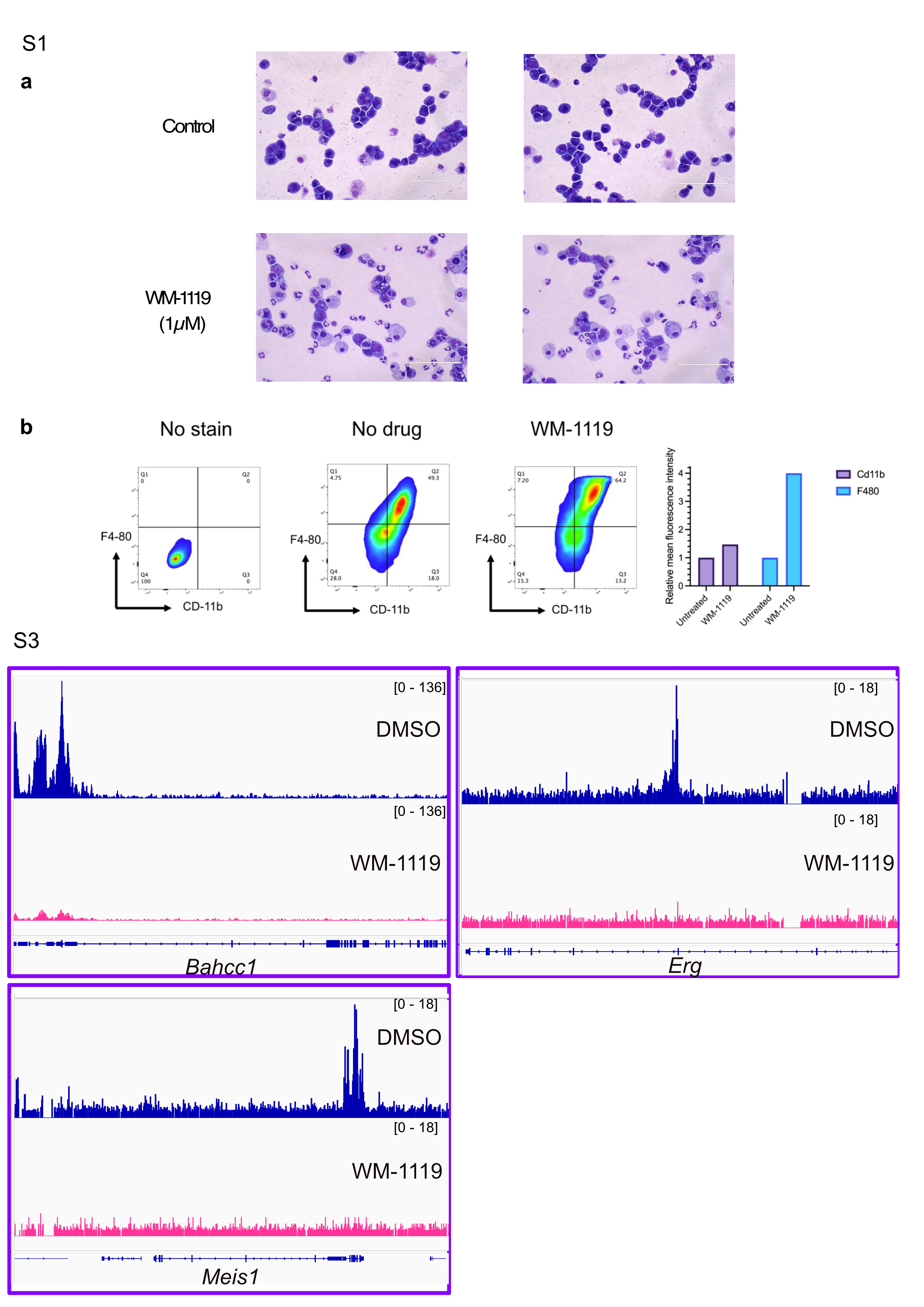

Supplement: Supplementary file 1 — Supplementary Material 1 [file 13045_2024_1610_MOESM1_ESM.tiff]

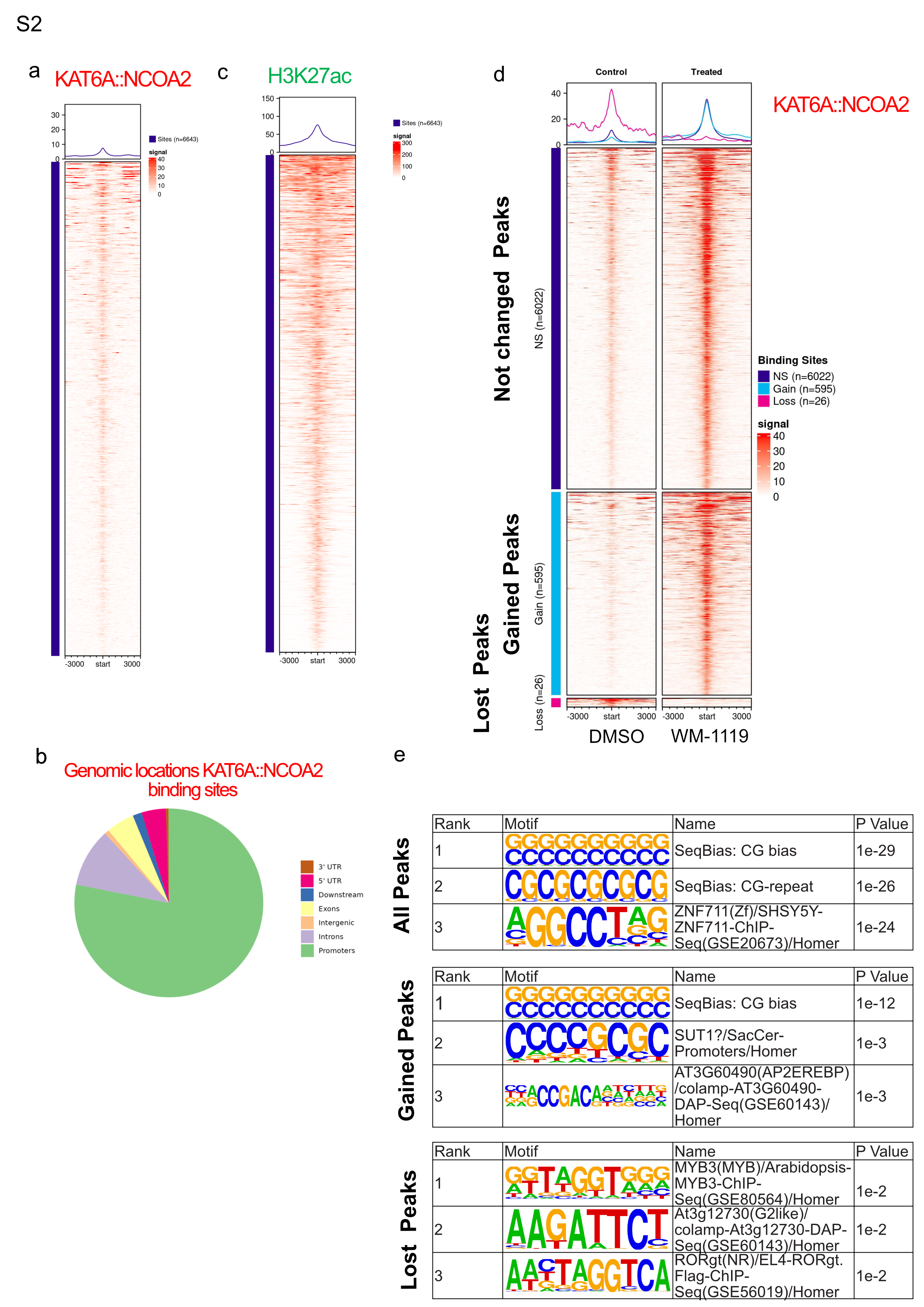

Supplement: Supplementary file 2 — Supplementary Material 2 [file 13045_2024_1610_MOESM2_ESM.tiff]

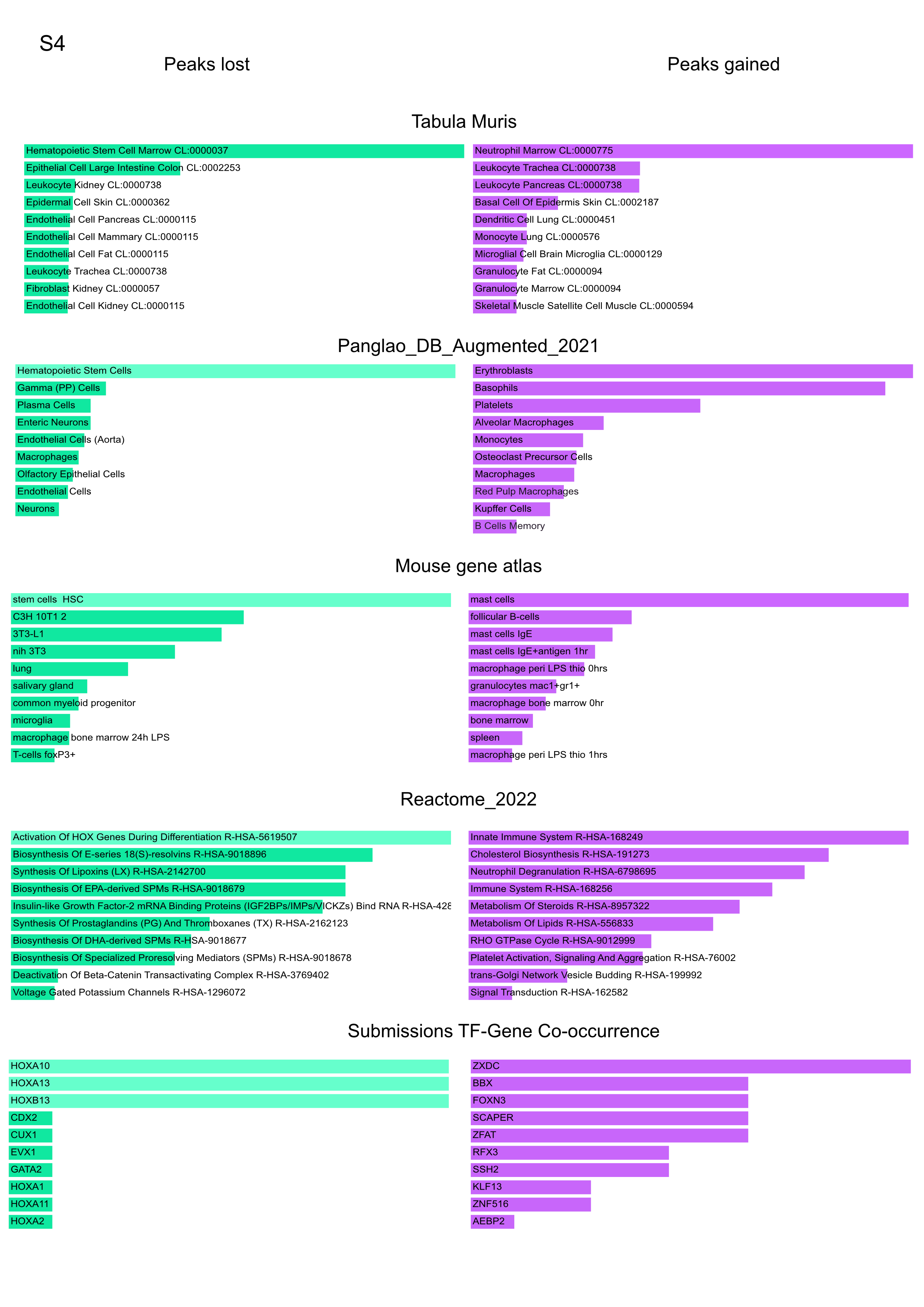

Supplement: Supplementary file 3 — Supplementary Material 3 [file 13045_2024_1610_MOESM3_ESM.tiff]

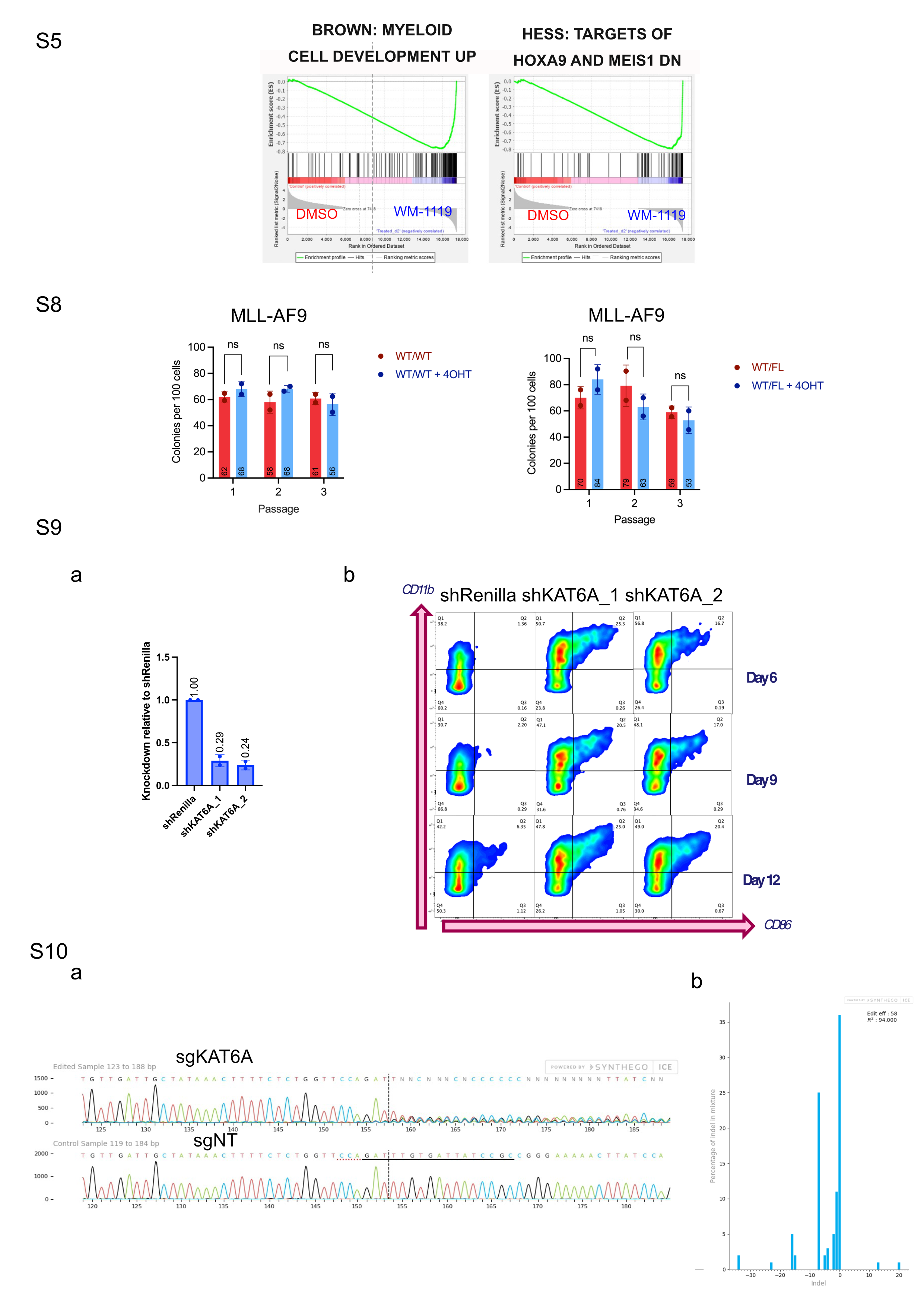

Supplement: Supplementary file 4 — Supplementary Material 4 [file 13045_2024_1610_MOESM4_ESM.tiff]

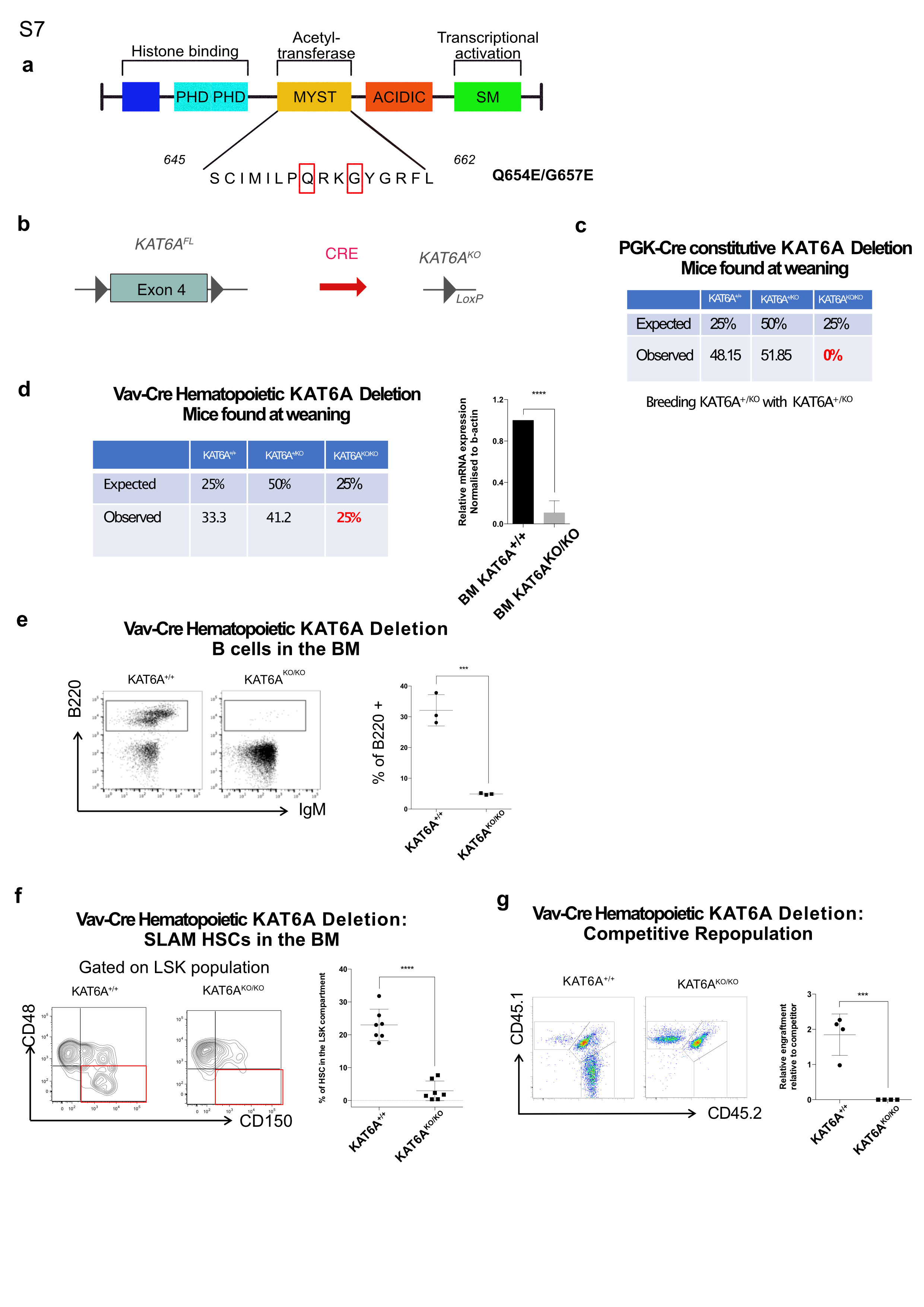

Supplement: Supplementary file 5 — Supplementary Material 5 [file 13045_2024_1610_MOESM5_ESM.tiff]

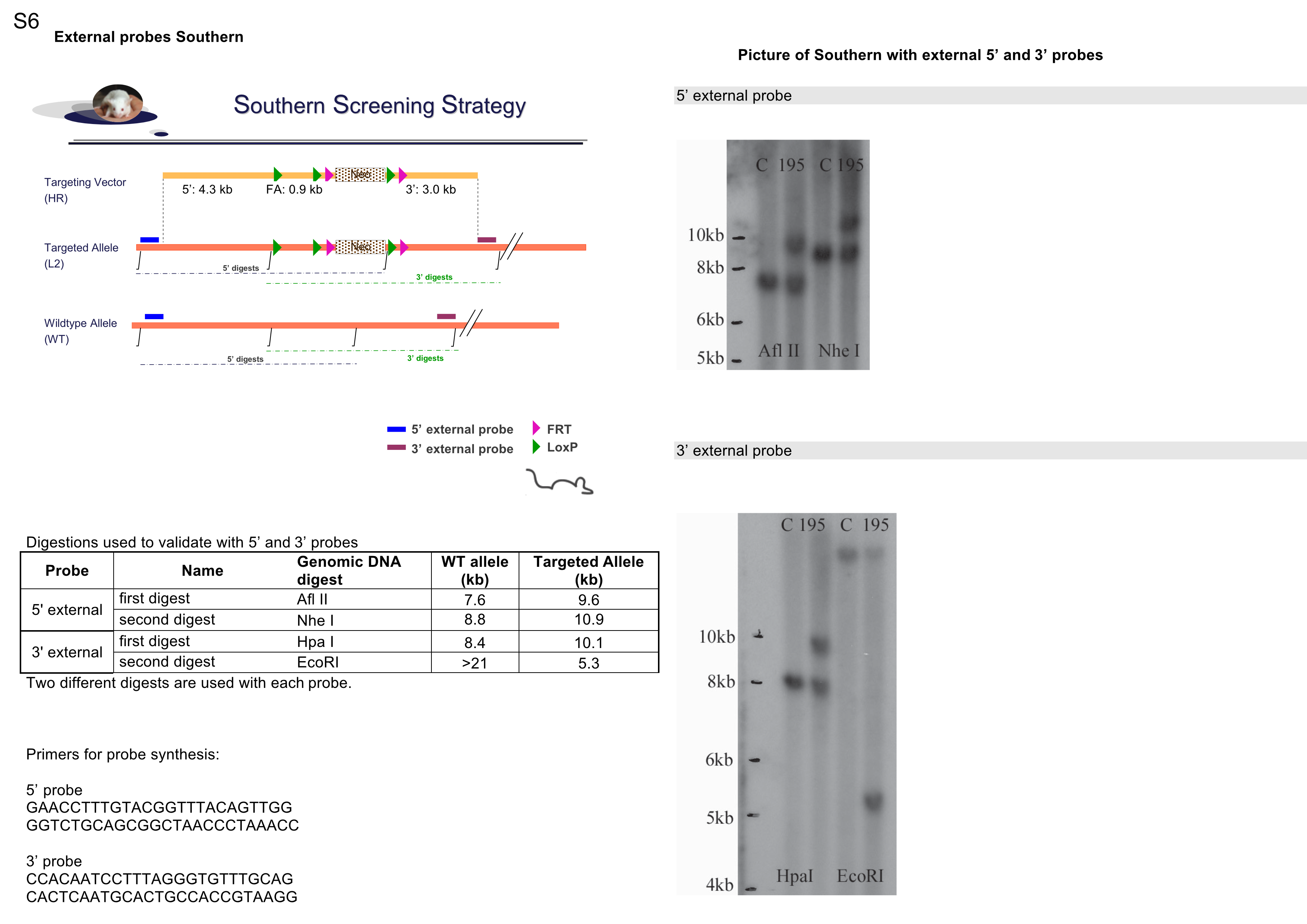

Supplement: Supplementary file 6 — Supplementary Material 6 [file 13045_2024_1610_MOESM6_ESM.tiff]

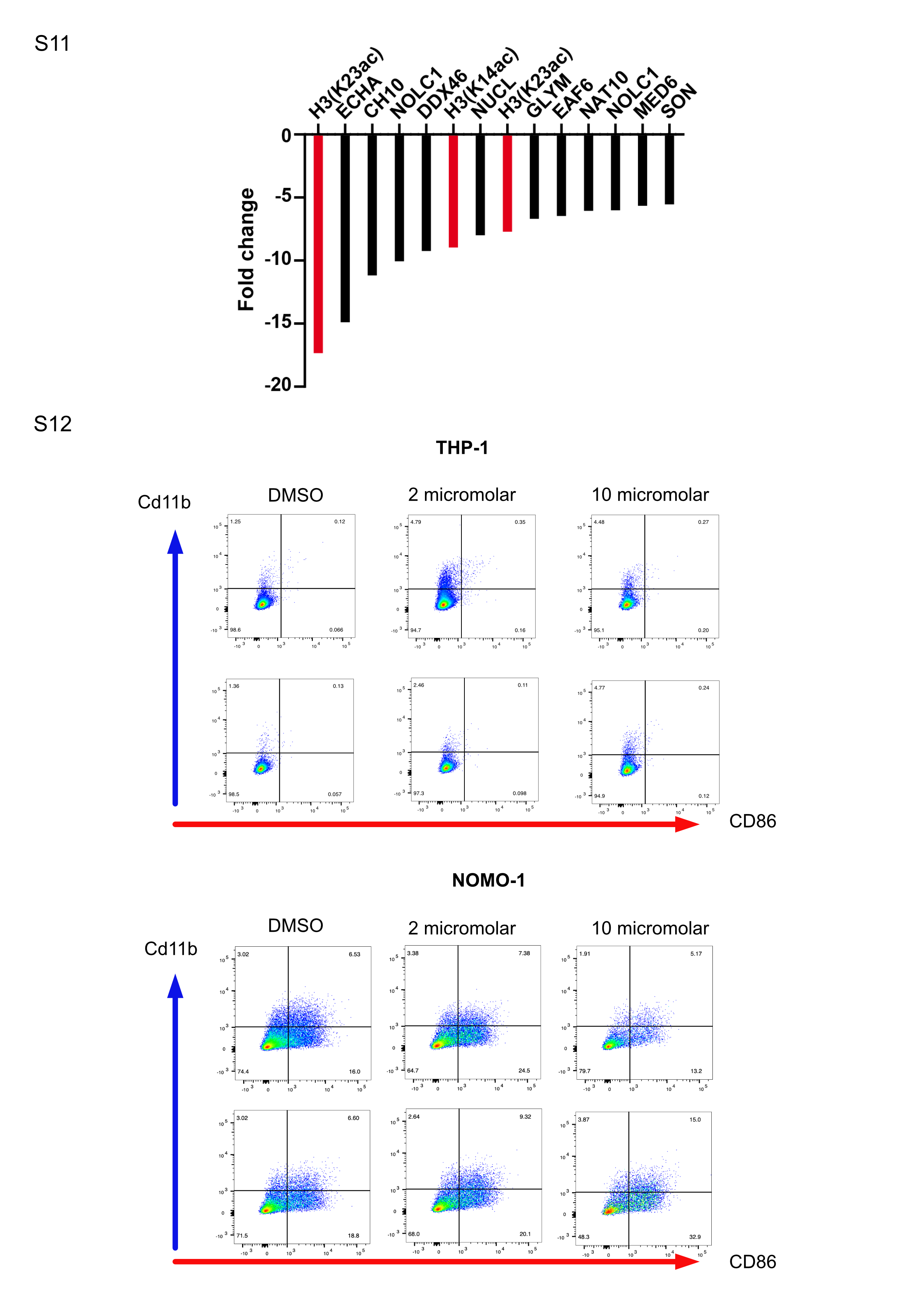

Supplement: Supplementary file 7 — Supplementary Material 7 [file 13045_2024_1610_MOESM7_ESM.tiff]
