# Supplementary material for: The small inhibitor WM-1119 effectively targets KAT6A-rearranged AML, but not KMT2A-rearranged AML, despite shared KAT6 genetic dependency
Source: J Hematol Oncol. 2024 Oct 8;17:91. doi: 10.1186/s13045-024-01610-0 (PMC11462755; doi:10.1186/s13045-024-01610-0)
Supplement: Supplementary file 11 — Supplementary Material 11 [file 13045_2024_1610_MOESM11_ESM.pdf]

**S1a**. Additional representative images of MT2 cells treated with DMSO or WM-1119 at 7 days

**S1b.** Flow plots and histogram of relative mean fluorescence intensities for MT2 cells at day 4 after being treated with 1 micromolar WM-1119 or DMSO every two days.

**S2a.** Ranked ChIP-seq signals for KAT6A::NCOA2.

**S2b**. Genomic locations of KAT6A::NCOA2 binding sites.

**S2c**. H3K27ac ChIP-seq signals for ranked KAT6A::NCOA2 binding sites.

**S2d**. Changes in KAT6A::NCOA2 binding sites upon WM-1119 treatment.

**S2e**.Homer motifs found at all, gained or lost KAT6A::NCOA2 binding sites.

**S3**. ChIP-Seq tracks at Bahcc1, Erg and Meis1 in MT2 cells treated with 2 micromolar WM-1119 or DMSO after 72 hours.

**S4**. EnrichR histograms showing top 10 hits by adjusted p value for selected "Cell Type", "Pathways" and "Transcription" gene sets for genes with loss of KAT6A::NCOA2 binding and genes with gain of KAT6A::NCOA2 binding.

**S5**. Leading edge plots for single cells at 48 hours of treatment showing a decrease in stemness and increase in myeloid development with WM-1119 treatment.

**S6.** Southern blot for generation of KAT6AFL/FL mice. KAT6A floxed allele was produced at the Institute Clinique de la Souris (ICS: Illkirch, France) under commission and design from L. Delva's laboratory. The targeted ES clones were identified by PCR

using external primers and confirmed by Southern blots with Neo and external 5' and 3' probes.

**S7a** Structure of the Kat6a Q654E/G657E mutant

**S7b** Structure of the Kat6a Floxed allele

**S7c** PGK-cre Kat6a deletion is embryonic lethal.

**S7d** Normal mendelian number of Kat6a knock out mice upon Vav-cre deletion. Vav-cre is active upon emergence of the first hematopoietic stem cell in the AGM.

**S7e** B220$^{+}$ cells are underrepresented in the bone marrow of mice with *Kat6a* deleted in blood system. Representative FACs histograms (left) and quantifications (right).

**S7f** LSK SLAM$^{+}$ HSC cells are underrepresented in the bone marrow of mice with *Kat6a* deleted in blood system. Representative FACs histograms (left) and quantifications.

**S7g** Deletion of Kat6a leads to the loss of repopulation capacity. FACS plots of blood 15 weeks after transplantation of WT competitor (CD45.1.2) and test population (either WT or MOZ deleted, CD45.2) in sub-lethally irradiated recipient mice (CD45.1) (left panel); quantification of the relative engraftment at weeks 15 (right panel)

**S8.** Controls colony counts from clonogenic assays assessing the effect of Cre recombination upon additional control genotypes, KAT6A$^{WT/WT}$ and KAT6A$^{WT/FL}$ (n=2 and two tailed unpaired t test, $p \geq 0.05$ (ns), 0.01 to 0.05 (*), 0.001 to 0.01 (**), 0.0001 to 0.001 (***), <0.0001 (****) for both).

**S9a** Histogram showing *KAT6A* expression by qPCR 3 days after induction of shRNAs against *KAT6A* relative to *KAT6A* expression 3 days after induction of an shRNA against *Renilla*.

**S9b** Representative flow plots showing the increase in CD11b and CD86 expression in cells in which KAT6A is targeted by different shRNAs (multiplicity controls) compared to cells in which KAT6A is not targeted (shRenilla)

**S10a** Sanger sequencing plots were used to Infer CRISPR edits for THP-1 cells. The dotted line marks the PAM site. An assigned knockout score of 56 indicated the proportion of indels that indicated a frameshift or were 21+ bp long.

**S10b** An Indel plot showing the "the inferred distribution of indels in the entire edited population of genomes" for THP-1 cells (Synthego).

**S11** Mass Spectrometry (PTMScan) for acetylated lysine proteins – graph showing Log Fold change of acetylated lysine proteins between none treated (DMSO) and 2 micromolar WM-1119 72 hours treated MT2 cells.

**S12**. Representative flow plots showing CD11b and CD86 expression at day 4 in THP-1 (n=2) or NOMO-1 (n=2) cells treated with DMSO, 2 micromolar or 10 micromolar WM-1119 on day 0.
